# Pro-cycling team cyclist assignment for an upcoming race

**DOI:** 10.1371/journal.pone.0297270

**Published:** 2024-03-04

**Authors:** Maor Sagi, Paulo Saldanha, Guy Shani, Robert Moskovitch

**Affiliations:** 1 Software and Information Systems Engineering, Ben-Gurion University of the Negev, Beer Sheva, Israel; 2 Israel – Premier Tech, Israel; 3 Population Health and Science, Icahn Medical School at Mount Sinai, New York City, New York, United States of America; Université de Lille: Universite de Lille, FRANCE

## Abstract

Professional bicycle racing is a popular sport that has attracted significant attention in recent years. The evolution and ubiquitous use of sensors allow cyclists to measure many metrics including power, heart rate, speed, cadence, and more in training and racing. In this paper we explore for the first time assignment of a subset of a team’s cyclists to an upcoming race. We introduce RaceFit, a model that recommends, based on recent workouts and past assignments, cyclists for participation in an upcoming race. RaceFit consists of binary classifiers that are trained on pairs of a cyclist and a race, described by their relevant properties (features) such as the cyclist’s demographic properties, as well as features extracted from his workout data from recent weeks; as well additional properties of the race, such as its distance, elevation gain, and more. Two main approaches are introduced in recommending on each stage in a race and aggregate from it to the race, or on the entire race. The model training is based on binary label which represent participation of cyclist in a race (or in a stage) in past events. We evaluated RaceFit rigorously on a large dataset of three pro-cycling teams’ cyclists and race data achieving up to 80% precision@i. The first experiment had shown that using TP or STRAVA data performs the same. Then the best-performing parameters of the framework are using 5 weeks time window, imputation was effective, and the CatBoost classifier performed best. However, the model with any of the parameters performed always better than the baselines, in which the cyclists are assigned based on their popularity in historical data. Additionally, we present the top-ranked predictive features.

## Introduction

Road bicycle racing has been an organized competitive team sport for 150 years [1]. In these races, teams of cyclists compete in a set of races held throughout the year, in which cyclists from the same team race together. The result of the race is dictated by the first team member to cross the finish line; hence, the entire team works towards bringing their best rider to the finish line as quickly as possible. Moreover, the sport of cycling has single and multiple race days, each with different characteristics that suit one type of rider or another. Although cycling is a very advanced and technological sport, in which quite some data is gathered, whether on the races and cyclists, or on their workouts sensors, and races, not much scientific research was published on the problem of assigning the right team of cyclists to a given race, based on all the available digital data. In this paper we introduce RaceFit, a decision support system that allocates cyclists to upcoming races [2]. RaceFit consists of certain classification methods including historical workouts and racing data. After extracting certain parameters from the raw data, statistical modeling is used to predict the probability that a cyclist fits to participate in a race. Additionally, we explore which features are most important in the coach’s decisions. We further elaborate on the motivation for this work and the contributions in this section and the Related Work section.

A top World Tour professional cycling team is usually composed of twenty-six to thirty cyclists, of whom six to eight compete in each race. The team’s sports directors and coaches determine the rider roster for each race [3, 4] based on many factors including the rider’s recent training and performance, and the race profile and conditions. Races vary substantially in location, terrain, and environmental conditions. Some are mountainous climbing events, while others are flat sprint finish events. The team’s performance group typically plans out the race schedule and designs a corresponding training plan for each cyclist to best prepare for the calendar.

Sometimes, there are roster changes, depending on the riders’ health and race readiness based on their ability to adequately prepare for the race.

Modern cyclists use a variety of devices to capture data such as GPS, power meters, heart-rate monitors, etc. This allows teams to record metrics like elevation gain, distance, heart rate, cadence, power, estimated energy expenditure, and overall workout duration. These data are typically uploaded to an online service such as Training Peaks [5] or STRAVA [6], which can be accessed by the performance staff of the team. These platforms allow a coach to prescribe training for each rider and to monitor his physical condition in training sessions and races. All these data are available to the performance group to make rider and race selection decisions. An additional source of information on races and rider performance is through the Pro Cycling Stats (PCS) website [7]. Currently, sports analytics is an emerging field of study, and cycling is no exception given the amount of data that the riders and team have access to. Some cycling studies have examined professional cyclists’ training loads and intensity characteristics, using direct and indirect measurements (i.e., utilizing heart rate) [8–10]; others compared the difficulty of races based on training loads, expressed by intensity and volume measured using heart rate [11]; and still others analyzed physical demands, fatigue, and power profile during a race ride [12–16]. Further, some studies have also attempted to assist coaches and athletes in making better tactical decisions for events in track cycling like Omnium races [17], while others focused on predicting race results [18, 19]. This project attempts to develop a decision support system to allocate cyclists in upcoming races [2]. Here, we introduce RaceFit, a support tool for cyclists’ fit for a race, consisting of certain classification methods including historical training and racing data. After extracting certain parameters from the raw data, statistical modeling is used to predict the probability that a cyclist will participate in a race in which they will have the best possibility of success. Understanding which features are most important in the coach’s decisions helps us to understand the coach’s decision process.

The contributions of this paper are the following: we introduce the problem of recommending cyclists for a race stage based on historical data, along with a dedicated dataset that we created for this task, and we propose a classification-based method for the recommendation of cyclists’ inclusion in a race stage, and provide a rigorous evaluation on the constructed real data.

## Related works

### Sport analytics

In recent years, much sports-related research has been conducted. Some studies have concentrated on the physiological domain, such as on estimating oxygen uptake during workouts [20]. Other studies focused on prediction tasks, for example, Hilmkil et al. [21] trained a model to predict the heart rate of a cyclist during a workout, and Kataoka et al. [22] focused on predicting power performance at the Tour de France 2017 grand tour using GPS data in addition to physical and environmental measurements. studies have attempted to predict cyclists’ performance in races using their ranking point results [23–25] or by analyzing tactical differences and their impact on gae performance, In this vein, Memmert et al. [26] explored different formations in soccer, Narizuka and Yamazaki [27] examined team behavior patterns in football games, while exchanging team structures.

#### Cycling analytics

In addition to Hilmkil et al.’s [21] work, in which the heart rate of a cyclist during a workout was predicted, other works related to cycling analysis examined physical measurements of cyclists during a ride. Vogt et al. [8] analyzed such measurements among professional road cyclists, and showed that intensity and workload estimation during a ride were better when measured with power, rather than with heart rate as was common before. Later, Vogt et al. [9] explored the power produced by cyclists during flat and mountain stages, and Lucía et al. [11] compared the difficulty of grand tours based on physiological loads. Erp et al. [10] compared male and female intensity and load characteristics in training sessions, and similar studies analyzed performance within cycling grand tours [12–16]. Additional works were done to assist coaches and athletes in making tactical decisions during races [17] or for predicting race results using machine-learning techniques [18, 19]. In our recent work [2], we modeled the coach decision-making process while using Training Peaks data for the Israeli pro cycling team Israel Premier Tech, and we discuss the importance of different factors in the decision process using the feature importance method.

### Recommendation systems

For many decades, humans have relied on recommendations regarding many decision-making processes in their daily actions. Over the years, recommendation systems have become involved in our lives oin different domains [28–31], especially in the past two decades. One reason such usage has skyrocketed can be attributed to the development of Netflix Prize [32–34]. These software tools give suggestions to a user based on the properties of the recommended entity or other similar users. Much information is gathered about a user and his habits, and the recommender algorithm produces a ranking list. The highest ranked objects on the list are those that are recommended to the user. The many types of recommendation techniques include Content-based filtering, based on the item recommended for properties, and Collaborative filtering, based on a user’s ratings.

Content-based filtering uses item features and rankings that a user liked previously. The items that are most similar to these are the ones recommended. [35] elaborate on this method and discuss user profiling and item representation, ranking algorithms along with real application examples. However, this method is limited by the fact that item properties are not always available. In contrat, Collaborative-filtering (CF) systems rely on the assumption that similar users would probably like the same items. These systems collect users’ rating data and consist of decisions on users’ preferred items. Nowadays, there are many domains using these systems, including music recommendation systems [36], movie and video contents [37, 38], and social networks [39]. In general, CF algorithms are grouped into approaches that are memory based [40], which maintain a user-item matrix of ratings to fetch data from, and those that are model based, which use model predictions from previous rating data [41]. Another approach is the Hybrid approach, which combines both of these types [42, 43]. Filtering methods can also be based on users (e.g., User-based filtering), where the similarity of other users is ranked to the user who is being queried [44], or this technique can be based on items (e.g., Item-based filtering), in which the similarity between itemsis calculated to target a user to other items similar to those that he liked previously [45].

### Sports recommendation systems

Sports research studies are typically motivated by how to increase chances of winning and turn in best performance. Some works discuss the potential and importance of improving athletic performance using machine-learning techniques [46, 47]. However, as far as we know, no studies have yet been done on cycling road recommendation systems. Therefore, in this work, we refer to recommendation systems in other sports such as running and soccer. While soccer player selection for a specific game seems to resemble the problem of cyclists’ allocation to races, there are significant differences between these tasks. For example, in soccer, everyone on a team attends each game, whereas in cycling, multiple races can occur simultaneously, and only several cyclists are required per race and cannot be replaced during the race. Berndsen et al. [46] presented methods to predict marathon results and suggested assisting athletes to create a personalized training plan to optimize their workouts. Smyth [47] proposed relevant applications using recommendation methods that focus on the endurance sports domain and personalized training plans. Berndsen et al. [48] suggested race recommendations for marathon runners. Their model predicts the finish time of a runner during a race based on real-time data and helps the runner make strategic decisions. Matthews et al. [49] discussed football results optimization, a work method that uses reinforcement learning to choose the ultimate team formation. They presented a feasible approach with top results, but the algorithm is complex, andteam managers do not understand and trust the decision process. Al-Asadi [50] discussed a decision support system that helps football team managers to select the best player to assign to a specific position on the field based on the player’s technical, physical, and mental skills. After selecting the ultimate formation of players, the system chooses the available squad (i.e., 4-3-3, 3-5-2, etc.) according to the formation of players that were previously chosen. The system predicts players’ skills growth and can help managers decide which players should be released, recruited, or kept.

An additional research domain related to sports recommendation systems is that of sports video content recommendations based on users’ consumption. Sanchez et al. [51] addressed the problem of consuming video content at the same time, specifically, that of Olympic games. The solution proposed was a distributed content-based system that runs on the client side. The system recommendations are based on the videos’ properties, and it computes the degree of interest for each attribute of that video on the client side and sends a recommendation to the server, which supplies content to users according to the recommendations. The recommendation process is transparent to the user and is executed without user demand. The main subject of the work focuses on computational problems for systems with large numbers of users—a problem not relevant to our purposes. The limitation of this approach is that it relies only on content in the decision process, in contrast to other popular well-suited approaches such as collaborative filtering, rejected here considering computational problems. Meng et al. [52] designed a video-based football real-time recommendation system, in which football players are recommended to users of the system based on their previous interests. The presented algorithm focuses on collecting system data algorithms. Their real-time tracking method, compared to GPS devices, is much more efficient and less costly, and provides close results. It uses Deep Learning models learning from multi-view videos. Because one of the challenges of real-time tracking is that a single angle of video provides poor performance, a proposed solution was to use videos from different angles and integrate them into the system into location coordinates.

Another problem that can be relevant in a recommendation task is the team replacement task. Li et al. [53] designed an algorithm that solves optimization problems based on graph theory. This algorithm takes into account team structure and member skills. The members of the team are represented as nodes, and the edge is the interaction between them. In tests of the algorithm on different domains, one of which was an experiment on NBA games, it outperformed its accuracy metric, but time complexity was a limitation. Thus, they used a pruning method for faster executions. In our work, race properties used complicate this task even more. In addition, because the cycling teams’ structure is similar in all cycling races, using this kind of model is unnecessarily complex.

## Methods

Here, we describe RaceFit, a framework for recommending selected cyclists from an entire team for a specific race based on previous races and workout data. We start by formulating the problem and continue with a description of RaceFit.

### Problem formulation

A *race* may include multiple *stages* having several properties such as distance, elevation gain, temperature, and more; a team of *cyclists* having properties such as age, weight, height; and the cyclist’s *workouts* described by properties such as duration, distance, power, and more. Given all the historical data of the teams’ races and cyclists’ workout data, we are interested in learning a recommendation model that will allow us, given a coming race and its properties, to rank a team’s cyclists according to their fit to the specific race, and to select the top k-ranked cyclists to participate in the upcoming race.

### RaceFit

**Fig 1:**
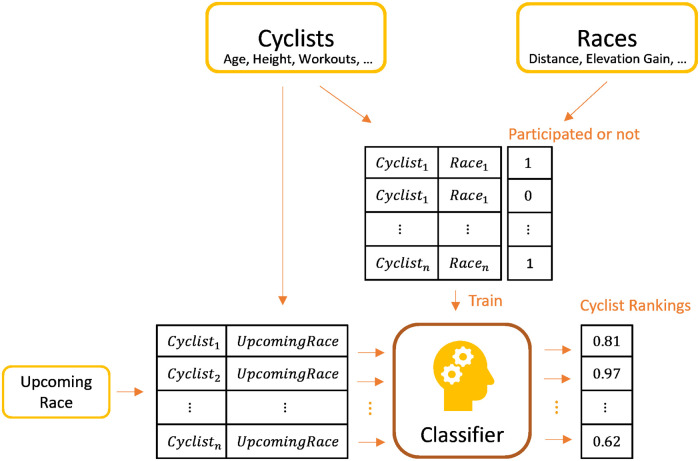
RaceFit, a block diagram of the binary classification-based recommendation framework. The classifier is trained on examples that consist of pairs of a race and a cyclist.

RaceFit is a novel classification-based recommendation method for cyclists’ assignments in a race. Our data consist of the team’s cyclists’ properties, workouts, and race stage data in which they participated. Thus, the task is to determine among the team’s cyclists who are the best to fit a race stage according to the upcoming race’s properties. We introduce here several approaches that we experimented with. One question is whether to recommend a collection of cyclists per race, or rather per each of its stages, and to aggregate the stage recommendations into a single recommendation per race. Another question is when recommending per stage and aggregating to a race, should stage recommendations be aggregated using the mean function or learned functions. The RaceFit ranking model framework is shown in Fig 1. There are several objects that are used: the cyclists, including their demographic data, races, and workouts; and the races, including those in which the team participated, and which cyclists participated in each race. The heart of the model is a binary classifier that is trained on records that contain data on pairings of cyclists and races, in which the label is whether a cyclist had participated in a specific race or not. Note, the records for each race include a record for each cyclist showing whether he participated in the race or not (the label). Then, in order to recommend a collection of cyclists from the entire list of the team’s cyclists for an upcoming race, the induced classifier is applied for each cyclist and classifies that record, producing the classifier’s score, which becomes a matching rank of the cyclist for the upcoming race pair. Thus, for each race, the classifier is applied multiple times for each of the team’s cyclists, and the top k cyclists having the highest scores are recommended as participants in the upcoming race.

Similarly, we introduce a classifier-based recommender that consists of examples of a pair of a stage and a cyclist. Thus, the classifier will be applied for each stage separately, ranking the cyclists for each stage. Later, all the ranks of each cyclist for the stages are aggregated into a single race rank recommendation, and the top cyclists are recommended for the upcoming race, as we will soon elaborate.

#### Model features

As described previously, the method’s input consists of two main types of entities: a cyclist and a race, which may include multiple stages. In one of the models, a stage is an entity of its own, thus, we refer to it as well.

The cyclist features are divided into two groups: their personal properties and aggregated features that consist of their workouts within a recent period of time that is defined as several weeks. Personal features may include properties such as age, height, and weight, and aggregated features such as the number of weeks before the upcoming race, the number of times the cyclist had raced with the current team before the upcoming race, etc. The aggregated features that consist of the recent weeks’ workouts may include features such as the total workout time, distance, elevation gain, calories burned during the workouts, etc. Typically, these will be aggregated by functions such as the average or the sum, for example, by the average distance in the workouts or the sum of the accumulated elevation within the workouts. Note that we are including one extra week, and the time point we consider is one week before the *race* date for the cyclists’ travel and preparation. For example, if the race takes place in the second week of February, and the time window size is two weeks, we will extract workouts from the two last weeks of January. The other entity we use as the method input is a race consisting of one or more stages, or a stage stands on its own. Stage properties such as total distance, elevation gain, stage ranking, etc. represent the stage as cyclist–stage input for the classifier, and other properties describing the stage are race features as metadata of the stage, such as the race class, the number of stages in the race, and the total distance or elevation gain in the stages of the race. Additionally, we are using another version of our method whose input is the cyclist–race pair.

The race entity features are the same as the stage features, and we use the average function to aggregate the stage features (i.e., average distance, elevation gain, and ranking of all stages in the race). The race features (class of the race, number of stages, etc.) stay without changes. Detailed features of all entities are available in the S1 Appendix.

#### Cyclist-Race recommendation

As was described more generally above, in order to learn the assignment of cyclists for a race, we learn a recommendation model that consists of a binary classifier that is intended to determine whether a cyclist will be assigned (or not) to an upcoming race. To accomplish this, we designed classifier examples consisting of cyclist and race pairings, whose label is whether the cyclist participated, or not, in the race.

**Fig 2:**
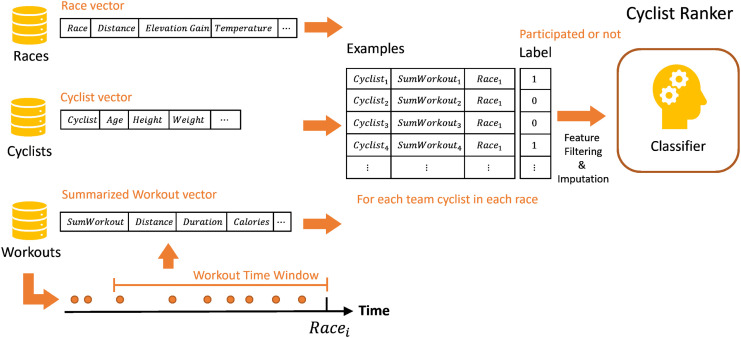
The training phase of the *RaceFit* given *race*, *cyclist*, and the *sumWorkout* summarized vector of workouts from the last weeks.

**Fig 3:**
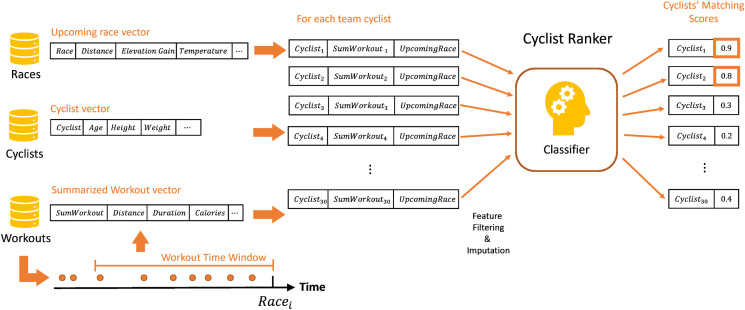
The recommendation phase of *RaceFit* given an upcoming *race* and the team’s cyclists along with their last workouts.

For greater clarity, the Figs 2 and 3 illustrate the training phase of the Cyclist–Race model, and then the recommendation phase, respectively. In the training phase, shown in Fig 2, a classifier is trained on examples whose features include pairs of a race’s features and each of the team’s cyclists represented by their features, and the label is whether they participated in the race or not. After the training table of the examples is constructed, several operations are performed because there are missing values in the data. First, filtering is performed, and features with missing values above a determined threshold are removed. Then imputation is applied, for which we used the mean imputation in which the average value of the feature is imputed.

The recommendation phase is illustrated in Fig 3. Once a classifier is induced, given an upcoming race and its features, a features vector is prepared for each cyclist, who is paired with the upcoming race’s features. Each of these vectors represents a cyclist on the team, and is given to the classifier, which provides a score that is the classification result and is considered as the probability of that cyclist’s participation in the race. The top cyclists, with the highest scores, are recommended for the race. Thus, for each upcoming race, the classifier is applied as a batch for each of the cyclists, and results in a list of ranks for each cyclist. However, while it makes sense to train a classifier according to the pairs of a race and each of the cyclists’ examples so that the classification is on the races, there are not many races, even over several years, as is the case here. Alternatively, since a race may include multiple daily stages, it is possible to train a classifier on pairings of a stage and each cyclist. This requires later aggregating the recommendation of the cyclists for the stages into a single score representing the recommendation for the race.

#### Cyclist–Stage recommendation

As was mentioned, another way to make a recommendation which is expected to provide more examples for the classifier and making it more robust and generalizing is classification based on each stage in a race, and eventually merging the scores of each cyclist to a various stage into a single score that represents the recommendation to a race.

**Fig 4:**
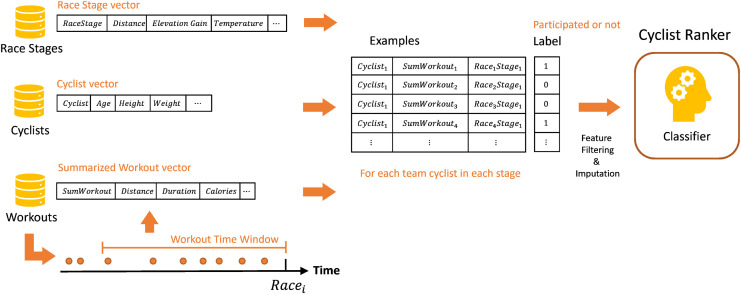
The training phase of *RaceFit* given *race*, *cyclist* and the *sumWorkout* summarized vector of workouts from the last weeks.

Fig 4 illustrates the training phase in the Cyclist–Stage recommendation. A race may include one day or multiple days, and each day is called a stage in the race. Here, the classifier is trained on examples that include the stage features, paired with each of the team’s cyclists; thus, an example is constructed for each stage for each cyclist, similar to the cyclist–race recommendation, but with the stages. The label of each example is whether the cyclist participated in that stage or not.

**Fig 5:**
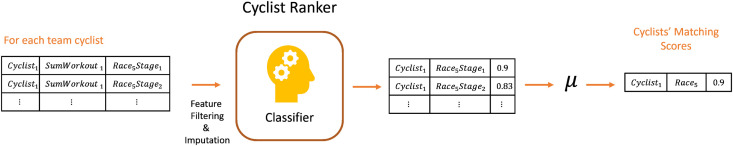
The recommendation phase of *RaceFit* given an upcoming *race* and the team’s cyclists, along with their properties and recent workouts.

The recommendation phase is illustrated in Fig 5. Here, similar to the cyclist–race model, for an upcoming race, for each of its stages, a record is constructed by pairing each of the cyclists on the team with their features. Thus, for each upcoming race, and for each of its stages, all the cyclists are paired with the stages, which are given to the classifier, which, in turn, produces the classification probabilities for each of the cyclists for each stage. Eventually, a cyclist–race recommendation score is calculated based on the average (which appears in the figure as *μ*) of the scores that the cyclist received for each of the stages. The top cyclists, having the highest scores, are recommended for the upcoming race.

#### Data preprocessing

RaceFit has several components that overcome challenges with the data or enable it to operate with various settings. First, since data are missing in various cyclist–stage examples, whether these are missing whole pairs of a cyclist–stage or specific features, we determine a dropping threshold based on the features that are removed. Thus, the *missing values threshold* is defined by the percentage of missing values in the feature. If the ratio of the missing values is above the threshold, then the feature is removed. After the relevant features are removed, in order to fill in the missing values, the framework uses imputation, for which various methods can be used. Additionally, another component of the framework is the classifier that can be used in order to perform the classification, as we demonstrate here with two options. The last parameter in the framework is the number of weeks prior to the given race stage from which features are extracted that summarize the cyclist’s workout performance.

## Evaluation

Our main goal in this work was to evaluate the performance of RaceFit, given its parameters, and to analyze which features of the cyclists, whether personal or those extracted from the workout data, and the race properties are most important in predicting the cyclist collection for a given race.

### Research questions

- Will using TP or STRAVA data result in different performances?
- Which method of classification-based recommendation will perform best, Cyclist–Race or Cyclist–Stage?
- Which performs best: with or without the use of imputation?
- Which of the modeling parameters will lead to the best performance: to include the workout time window size and which classifier?

### Cyclists’ workouts and race dataset

To perform the analysis and evaluate the proposed model, we created our database based on cyclists and races of PCS data and team workouts; Israel-Premier Tech (IPT), Jumbo-Visma, and Groupama-FDJ from STRAVA, and IPT workouts from Training Peaks (TP). Workout data from TP and STRAVA include the team’s cyclists’ workout and race data that are uploaded from wearable devices, such as a heart rate monitor and cycling computers. The physical measures include continuous measurements ofheart rate, cadence, power produced, speed, estimated calories burned, and energy consumption. The TP and STRAVA properties are very similar. In STRAVA, there is location data, and the training measures are training load and intensity, while in TP, there are TSS and IF training measures and missing location. Another source of information is the PCS data that include cyclists’ personal details such as age, height, weight, and nation; information about the race stages, including the location, date, UCI race classification, cyclist’s specialty rankings (i.e., sprint or climbing skills); and stage type: classic stage race, prologue, one-day race, or time trial. There are 583 cyclists in our cyclist dataset, including 94k TP workouts and 373k STRAVA workouts from late 2016 to 2022, and 6k races from 2010 to 2022. The dataset is accessible and can be downloaded online [54].

### Experimental plan

In the experiments below, we use the following protocol: for each race *r*, in order of occurrence, all the data prior to *r* are used as the observational data, including both the workout data and the race data within this period of time. We construct a separate model for each race. The model for the first race, hence, has no historical training data, and selects cyclists randomly, and in the next races, the training data are increased (with more races). The model accuracy is expected to improve for later races, as more data are included from races prior to it which are used for training. We train the various models and then predict which cyclists will be recommended for the race *r*. Following preliminary experiments, for the workout data, we focus on a 5-week window prior to the race, ignoring the last week before the race. This is because we assume that the final coach decisions will have been made prior to that week to allow the cyclists time to prepare and travel to the race location. We evaluate each model separately and refer to the mean results over all models, and describe here each of the experiments and their main purpose, followed by a description of the model’s parameters and their values with which the experiments are made.

#### Experiment 1—Cyclist–Race on TP vs. STRAVA

In the experiments below, we use the following protocol: for each race *r*, in order of occurrence, all the data prior to *r* are used as the training set, including both the TP and STRAVA workout data and data from the previous race. We construct a separate model for each race. The model for the first race, hence, has no historical training data and selects cyclists randomly. We expect the model accuracy to improve for later races. We train the various models on race data and then predict which cyclists will be chosen for the race *r*.

#### Experiment 2—Cyclist–Stage on TP vs. STRAVA

In this experiment, we use the same protocol as in Experiment 1, only here, we train the models on cyclist and stage data (not directly on race data), predict the probability of cyclist *c* participating in stage *s*, and then average for each cyclist *c* in each race *r* all the probabilities, deriving the score for the cyclist in the race.

#### Experiment 3—Cyclist–Race all teams on STRAVA

In the experiments below, we use the following protocol: for each race *r*, in order of occurrence, all the data prior to *r* are used as the training set, including both the STRAVA workout data and data from the previous races. We construct a separate model for each race. The model for the first race, hence, has no historical training data and selects cyclists randomly. We expect the model accuracy to improve for later races. We train the various models on race data and then predict which cyclists will be chosen for the race *r*.

#### Experiment 4—Cyclist–Stage all teams on STRAVA

In this experiment, we use the same protocol as in Experiment 3, only here we train the models on cyclist and stags data (not directly on race data), predict the probability of cyclist *c* participating in stage *s*, and then average for each cyclist *c* in each race *r* all the probabilities, deriving the score for the cyclist in the race.

### Experimental setup

The results we report here are based on RaceFit’s parameter settings, including all the parameter values that were tried. Additionally, we describe the baseline methods that were used for comparison to verify that the model learning is meaningful.

The parameters of RaceFit that we experimented with include the imputation method, number of weeks prior to the race that the summary of workouts consists of, and type of classification method. For the missing values, the best-performed thresholds used were: 40% for Experiment 1 and 3, based on cyclist–race method, and 60% for Experiment 2 and 4, based on cyclist–stage method. After their removal, the runs with or without imputation using the Scikit-learn [55] package included SimpleImputer with the default parameters. This imputation method is based on imputting missing values with the mean value. We also experimented with various time windows of 3, 5, and 7 weeks prior to the race for the summarized workout data. Finally, several classifiers were used including: CatBoost [56], Random Forest [57], Decision Tree [58], and Logistic Regression [59], which provide a good representation of various approaches for classification.

We defined two baselines that are based on the “popularity” of the cyclists in their appearance in races. Thus, a cyclist who was assigned to races most frequently before an upcoming race (the number of races the cyclist participated divided by the number of races the team participated in until the upcoming race) will be highly recommended. We had two versions of this baseline: one in general, and the second within the continent of the upcoming race. Thus, in the baselines, the assignment of cyclists to an upcoming race was based on their previous frequency of appearance in races, assuming they would be a default choice.

### Evaluation metrics

To measure the accuracy of RaceFit, given the list of recommended cyclists ordered by the decreasing predicted likelihood of participating in the race, we take the top *i* cyclists in the recommended cyclists’ list by RaceFit, and compute the *precision*@*i* and *recall*@*i* metrics, defined in Eqs 1 and 2, respectively. *Cyclists Raced* refers to the actual cyclists who participated in the tested race, and the *top i Recommended* refers to the top *i* recommended cyclists who were ordered by RaceFit.

$$\begin{array}{c} precision@i=\frac{\left|Cyclists\;Raced\;\cap \;top\;i\;Recommended\right|}{\left|top\;i\;Recommended\right|} \end{array}$$ (1)

$$\begin{array}{c} recall@i=\frac{\left|Cyclists\;Raced\;\cap \;top\;i\;Recommended\right|}{\left|Cyclists\;Raced\right|} \end{array}$$ (2)

Precision and recall are most appropriate here because this can be considered as a one-class prediction problem, i.e., we are only interested in correctly predicting which cyclists will participate in the race.

While in many applications, precision—the portion of correct predictions in the predicted list is most important—in this application, the number of correct predictions of cyclists who participate in a race is very low; hence, recall seems more expressive. Moreover, it is likely that when the upcoming race requires *n* cyclists, the coach will ask the framework for at least *n* recommended cyclists; however, the coach typically will require additional *k* recommended cyclist backups in case any of the top favored cyclists are not able to participate. Hence, we also compute *recall*@(*n* + *k*) for *k* = 0, 1….

### Results

The results of our evaluation of the data of Israel-Premier Tech, Jumbo-Visma, and Groupama-FDJ teams are presented here. First, we present a comparison of the performance of RaceFit on STRAVA data versus TP data for the IPT team (we had access to TP data only for this team). This section provides full results for experiments 1 and 2, and partial results for experiments 3 and 4, presenting only the averages, while the detailed and comprehensive outcomes for each team are available in the S2 Appendix.

#### Experiment 1—Cyclist–Race on TP versus STRAVA

**Fig 6:**
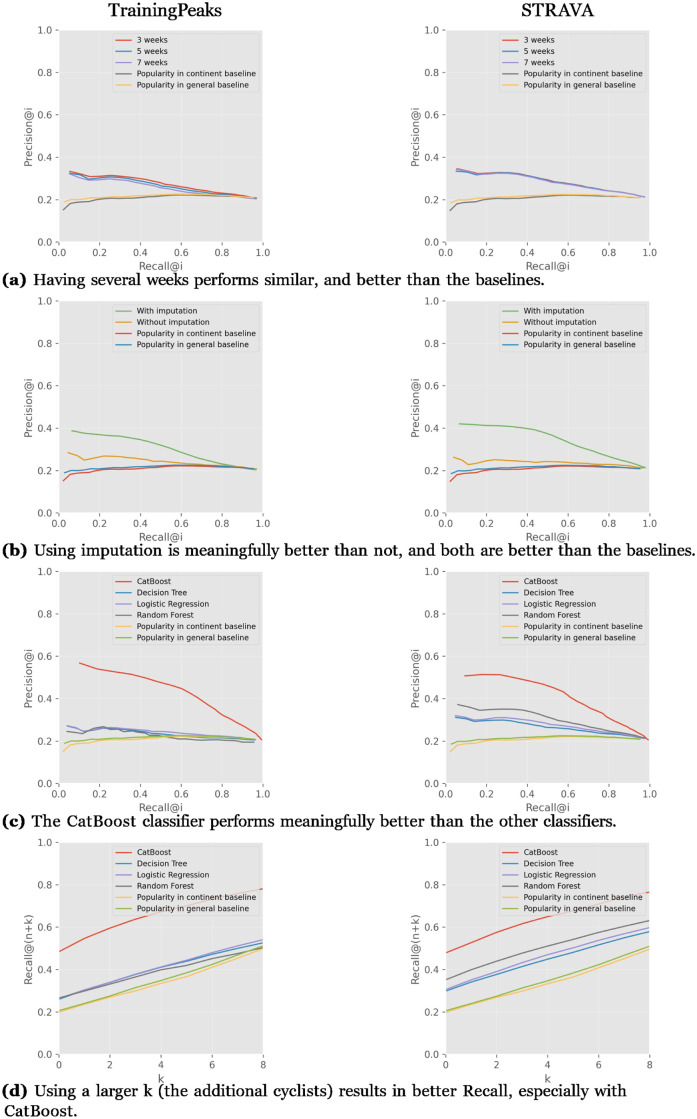
Generally using the TP and STRAVA data performs similar, and using imputation and the CatBoost performs best.

We present here the results for the IPT team on both STRAVA and TP for comparison, using the cyclist-race model. Fig 6a show the results for 3-, 5- and 7-week time windows of workout aggregation, in comparison to the baseline. The results for both data sources are quite similar, also for the sizes of time windows, and they perform much better than the two popularity baselines.

Fig 6b show the impact of the use of imputation. We can clearly see that using imputation performs better than not using it, and also much better than the popularity baselines.

Fig 6c and 6d show the performance on both data sources when using CatBoost, Decision Tree, Logistic Regression or Random Forest classifiers. Using CatBoost meaningfully outperforms the other classifiers in TP and STRAVA. However, the performance on TP is somewhat higher than on STRAVA. Following CatBoost, Random Forest performs best, while also better on STRAVA, and all the classifiers perform better than the popularity baselines.

Fig 6d show the *recall*@(*n* + *k*), where *n* is the number of cyclists who actually raced in a race, and *k* is the extra gap of cyclist recommendations that the system will recommend to the coach. For *k* = 2, the best classifier, CatBoost, the *recall*@(*n* + 2) is 0.6 for both data sources. Choosing *k* = 2 makes sense to use when the team coach has two extra recommended cyclists, leaving him space to choose from.

#### Experiment 2—Cyclist–Stage on TP versus STRAVA

**Fig 7:**
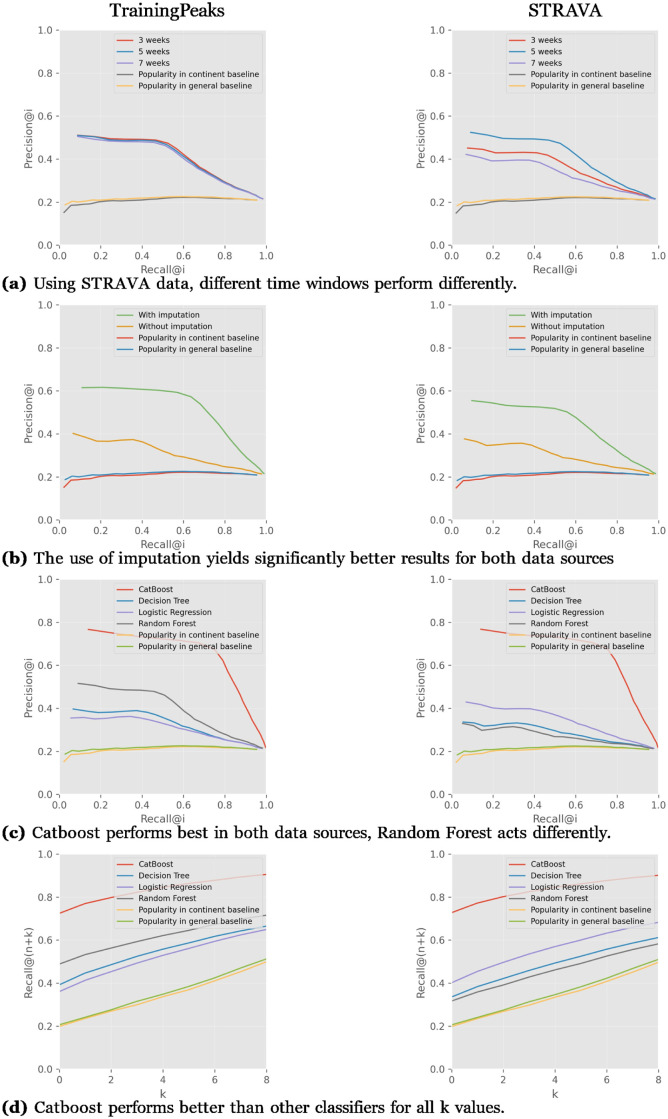
Using TP and STRAVA data with imputation and Catboost classifier, outperform other parameters.

The second experiment is similar to the first, but applies the cyclist–stage modeling on the IPT team’s data both from STRAVA and TP for comparison. Fig 7a show the aggregation of 3-, 5-, and 7-week workout time windows compared to the baselines. In the TP graph in Fig 7a, the results of all the time windows are quite similar and meaningfully better than the comparison baselines. In Fig 7a, in the STRAVA experiment, 5-week window performs better than at 3 and 7 weeks. For all the time windows tested, the results outperform the popularity baselines.

Fig 7b show the meaningful improvement when RaceFit uses imputation both with TP and STRAVA data. The performance without imputation of both data sources is better than the popularity baselines.

Fig 7c and 7d show the performance of both data sources when using the four types of classifiers: CatBoost, Decision Tree, Logistic Regression, and Random Forest classifiers. The CatBoost classifier on both TP and STRAVA data performs significantly better than the other classifiers. Random Forest performs quite better than Logistic Regression and Decision Tree in TP, but in STRAVA, it does not. The Decision Tree performs worse than other classifiers in STRAVA, and in the TP graph, Logistic Regression performs worse than the other classifiers. In both graphs, popularity baselines performed worse than RaceFit. Fig 7d show the *recall*@(*n* + *k*), where *n* is the number of cyclists who actually are recommended to a race, and *k* represents the spare cyclists that the system will recommend to the coach. For different values of *k*, the CatBoost meaningfully outperforms the other classifiers and popularity baselines. Random Forest on the TP data performs better than Logistic Regression and Decision Tree.

#### Experiment 3—Cyclist–Race all teams on STRAVA

**Fig 8:**
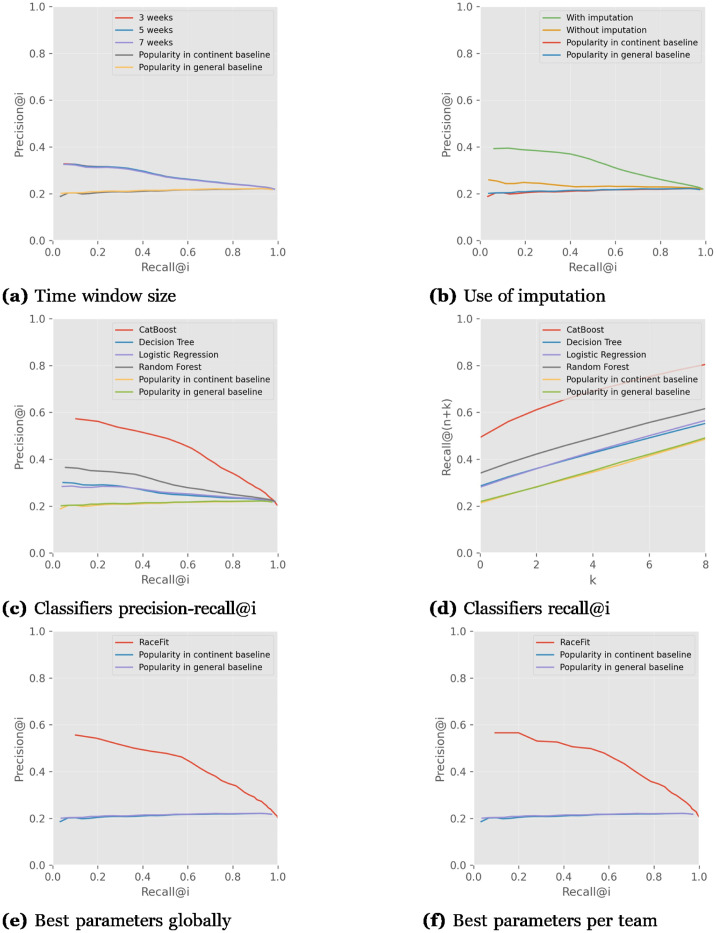
Comparison of the different parameters on average for all teams, and best run parameters results on average. Catboost and the Imputation use improve the model performance significantly.

Since in the previous experiments it was seen that the performance was quite similar whether using TP or STRAVA, we extend our experiments to two more pro-cycling teams, Jumbo-Visma and Groupma-FDJ, whose TP data was not accessible. We describe here the use of the cyclist–race modeling results after applying them to each of the teams, and their average to demonstrate the general performance. Fig 8a presents the performance of recent workouts over multiple time windows, represented in each curve using the cyclist–race model, in comparison to the baselines. The charts for all the teams can be found in the S2 Appendix, on average, Fig 8a the performance is quite the same for any of the time windows, and the use of RaceFit outperforms the popularity baselines. Note that these results are the mean of multiple modeling choices, including the use of not using imputation and data removal, as well as several classifiers, whose results are relatively not so good, but this does show the overall effect of using several weeks. In the later experiments, we will show the results with the best performing values.

Fig 8b shows the performance when using imputation or not, which clearly shows that using imputation was much better for all the teams. However, without imputation, the performance is still better than that of the popularity baselines.

Fig 8c shows the performance using several classifiers, in which all perform better than the baselines, while the use of the CatBoost classifier performs meaningfully better in both metrics. Random Forest is second best after CatBoost, and on average for all teams, the Decision Tree classifier is slightly better than Logistic Regression.

Fig 8d shows the *recall*@(*n* + *k*), in which *n* is the number of cyclists who are required to participate in an upcoming race and *k* is the number of spare cyclists the system recommends for the upcoming race. We see that when requesting the exact number of required cyclists (k = 0) for the upcoming race, CatBoost correctly recommends about 50% of the cyclists who in fact participated in the race. The Catboost outperforms the other classifier, than the Random Forest, and the Decision Tree performs better than Logistic Regression for *k* values smaller than four.

Fig 8e shows the performance of the best-performing parameters on average for all teams, based on the previous analyses. The best time window size on average over all the teams is 3 weeks, use of imputation improves the results, and CatBoost outperforms the other classifiers. The parameter results on average over the teams are not identical to the best-performing parameters for each team. In Fig 8f we present the performance of each team according to their best parameters’ values.

Fig 8f presents the model’s parameters with the best-performing values for each team individually, on average.

#### Experiment 4—Cyclist–Stage all teams on STRAVA

**Fig 9:**
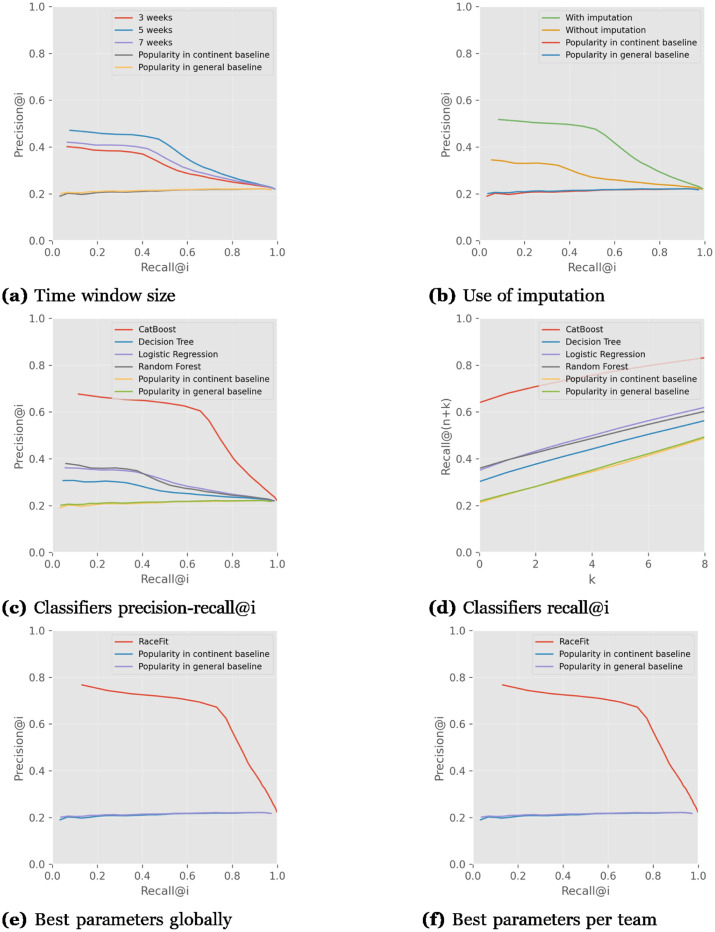
Comparison of the teams results on average using Cyclist–Stage algorithm. 5-weeks window, using imputation and Catboost classifier provide better results than other parameters.

We describe here the results of the cyclist–stage experiment on the various teams’ on STRAVA data. Fig 9a presents the performance of the different time windows in weeks. For all the teams, the use of a 5-week time window yields the best performance. On average, the 3-week window performs worse than the 7-week window. For all time windows, RaceFit performs better than the popularity baselines.

Fig 9b shows the advantage of using imputation. In all of the teams, the use of imputation improves performance. However, even without imputation, the performance is better than the popularity baselines.

In Fig 9c, the results of the classifiers that were evaluated are shown. For all the teams, the CatBoost classifier outperforms the others. On average, Random Forest is the next best performing classifier, Random Forest performs similarly to Logistic Regression.

Fig 9d shows the *recall*@(*n* + *k*) for the cyclist–stage version of RaceFit, in which *n* is the actual number of cyclists recommended and *k* is the number of spare cyclists recommended, for the coach to choose from if needed. The CatBoost classifier yields the best performance for all of the teams, followed by Random Forest. For all the teams, the baselines perform more poorly than the RaceFit algorithm.

Fig 9e presents the best-performing parameters on average over all teams’ results. The best performance achieved by window size is the 5-week window, the use of imputation to fill missing values, and CatBoost as a classifier.

Fig 9f shows the results of the best parameters chosen for each team individually. The best time window for all the teams is the 5-week window. The best classifier for all teams is CatBoost, and use of imputation was effective for all as well. Looking at the results in Fig 9f and the parameters that worked best for all teams on average, the results perform quite similarly; thus, it is reasonable to use the parameter values that work for all teams on average.

In order to demonstrate the most important features used, we implement two approaches for feature importance: data-driven methods, including Relief [60], Information Gain and Chi-Square using the Scikit-learn library [55] implementation; CatBoost’s feature importance [61], and the SHAP Tree Explainer values [62] are used. In the following section, we discuss the results of the feature importance, whose bar plots with detailed values can be found in the S3 Appendix.

We divide the features into three groups: first, the cyclist features including general and statistical data, next, the workout features, and last, the race features.

## Discussion and conclusions

The use of analytics in various fields of sport is becoming more common especially for professional athletes and teams. While cycling have already years of sensory use, such heart rate, and the bikes metrics, including the cadence, power, speed, and other, there were no much relevant studies in the literature. In this paper, we described the problem of pro-cyclist assignment to races, which as far as we know, is investigated for the first time here using a data-driven approach. For this purpose, we created a large and meaningful dataset of professional cycling teams that consists of races and stages data, cyclists and their participation and performances, as well as workout data. Data was collected from two distinct sources: Training Peaks and STRAVA. The Training Peaks data exclusively pertains to the Israel—Premier Tech team, whereas the STRAVA data, is publicly accessible, and includes additional professional teams. A comparison of the data specifically for the Israeli team reveals a strong consistency between Training Peaks and STRAVA data. This similarity is likely attributed to the practice of cyclists uploading their workout data to both platforms. We introduced RaceFit, a recommendation support system for cyclist assignment in an upcoming race based on its properties, and introduce two approaches, both consisting of a binary classifier. In the first approach, the input for the classifier consists of a single cyclist–race pair, which is trained on whether a cyclist is assigned to a race or not. The second approach consists of a binary classifier as well, but the input here consists of all the cyclist–stage pairs for all stages in a race. The classifier output is aggregated using the average function into the final score. The cyclist-race pairing appears more intuitive, as our method predicts the scheduling of cyclists for races. Unfortunately, however, due to the limitations of a relatively small dataset (while the biggest available according to current sources), the cyclist-race experiments showed lower performance when compared to the cyclist–stage experiments. It can be reasonably inferred that this difference arises since there are less examples of races than stages (some races consist on multiple stages) the model was trained on. In our research questions, we asked if there are any differences in the results of TP’s based data which is to some extent more complete, and STRAVA data. In the first experiment of cyclist–race method, and the second experiment of cyclist–stage method, we saw that the results were quite similar for both data sources. Thus, we could extend our experiments to more teams for which we have only their STRAVA data. Looking at the second question about which method is better—cyclist–race or cyclist–stage, the cyclist–stage performance was better as shown in the third and fourth experiments. Additionally, the third question referred to the model’s best parameters, in which all experiments have shown that the use of imputation improved the performance meaningfully. Then, the different time windows in the third experiment performed quite the same, while in the fourth experiment, the 5-week time window outperformed the others. Last question to answer was about the classifier, which in both the third and fourth experiments the CatBoost classifier performed best.

The first two experiments were performed only on IPT data, in which we had both TP and STRAVA data, showing that the results were quite the same with both data sources. Additionally, these experiments showed that RaceFit, when used for cyclist–stage assignment, performs better than assignment to races with the use of effective imputation, while using the CatBoost classifier, which was also better than the popularity baseline. Using STRAVA data, the third and fourth experiments were performed on three professional cycling teams: IPT, Groupama-FDJ, and Jumbo-Visma. The results for the three teams are similar, the window size that performs best is 5 weeks, use of imputation, and CatBoost as a classifier. In all experiments, RaceFit provides better performance than the popularity baselines.

On average, the performance of the three teams surpassed the popularity baselines, a common practice in athlete selection across various sports. In cycling, unlike many other sports, a season consists of a relatively large number of races, necessitating the scheduling of less popular or successful cyclists. After races, cyclists require rest to recover from the significant physical strain experienced during the events. Consequently, a consistent strategy of scheduling only the most successful and popular cyclists for all races may compromise the overarching goals of the team. Nonetheless, given the diverse impact that races have on team rankings, it is sensible for coaches to customize cyclist selection, particularly in races of lesser significance.

A limitation of this study is the data itself, which was downloaded from STRAVA, in which some cyclists do not appear, or do not share all their workouts. However, we downloaded many teams, and in evaluation, we focused on the teams whose data included most of the cyclists, their workouts and races and were quite complete. Lastly, in the feature importance analysis, we noticed that data-driven methods give more weight to the workout features than the incorporating model methods. In future work, it will be interesting to explore algorithms with different structures such as modeling the problem as assignment tasks for the whole year and evaluating it in this granularity, as well as incorporating the potential points accumulation annually as the utility. Recommendation systems are designed to influence user behavior, in our case, the coach. However, our goal is to use data from teams, and learn their decision making to learn recommendations that lead to successful performance.

## Supporting information

S1 Appendix Detailed features. Features of Cyclist, Race, and Workout entities with descriptions.
(PDF)

S2 Appendix Detailed results. Full results of Experiment 3,4 for Israel-Premier Tech, Groupama-FDJ, and Team Jumbo-Visma.
(PDF)

S3 Appendix Feature importance analysis. Results of the feature importance analysis with description.
(PDF)
