# Supplementary material for: Pro-cycling team cyclist assignment for an upcoming race
Source: PLoS One. 2024 Mar 4;19(3):e0297270. doi: 10.1371/journal.pone.0297270 (PMC10911621; doi:10.1371/journal.pone.0297270)
Supplement: S1 Appendix — Features of Cyclist, Race, and Workout entities with descriptions. (PDF) [file pone.0297270.s001.pdf]

Appendix 1 - Detailed features

Here, you can see the features, along with a description of the entities in our work. Table 1 presents the features of cyclists taken from the PCS, Then Table 2 presents the features of Training Peaks and STRAVA, andTable 3 shows the race features from the PCS.

Table 1. Pro-Cycling Stats Cyclist features

Feature	Description
Cyclist weight	
Cyclist height	
Cyclist age	
Cyclist week count on team till upcoming race	Number of weeks since cyclist joined the team
Cyclist total race count till upcoming race	Number of races cyclist raced since joining the team
Cyclist total race count last year till upcoming race	Number of races a cyclist raced during the last year
Cyclist total race count in race continent till upcoming race	Number of races a cyclist raced in the upcoming race's continent since joining the team
Cyclist race rate till upcoming race	Number of races cyclist raced divided by the number of team
Cyclist race rate in continent till upcoming race	Number of races cyclist raced in race's continent divided by the number of team races in the upcoming race's continent
Cyclist popularity ranking till upcoming race	Ranking of cyclist race rate compared to other cyclists on the team
Cyclist popularity ranking in continent till upcoming race	Ranking of cyclist race rate in continent compared to other cyclists on the team
Cyclist week count since racing till upcoming race	Number of weeks since cyclist last raced
Cyclist last race distance from upcoming race	Distance in kilometers from the last race cyclist raced to the upcoming race
Cyclist sprint points till upcoming race	Cyclist sprint ranking
Cyclist climber points till upcoming race	Cyclist climbing ranking
Cyclist one-day race points till upcoming race	Cyclist ranking in one-day races
Cyclist general classification points till upcoming race	Cyclist general classification ranking
Cyclist time trial points till upcoming race	Cyclist ranking in time trial stages

Table 2. Training Peaks and STRAVA Cyclist features

Feature	TP	STRAVA	Description
Workout last continent		V	

Table 2. Training Peaks and STRAVA Cyclist features

Feature	TP	STRAVA	Description
Workout total distance	V	V	Workout total distance in pre-defined time window
Workout total duration	V	V	Workout total duration in pre-defined time window
Workout avg hr	V	V	Workout heart rate average in pre-defined time window
Workout total calories	V	V	Workout total calories burned in pre-defined time window
Workout total TSS	V		Workout total TSS in pre-defined time window; TSS is training intensity measure considering normalized power, intensity factor duration
Workout avg IF	V		Workout average intensity factor (IF) in pre-defined time window; indicates how hard the workout was in relation to cyclist's fitness
Workout avg intensity		V	Workout average intensity in pre-defined time window; showing how difficult a workout was
Workout avg training load		V	Workout average training load in pre-defined time window; determining how much load put on cyclist's body during the workout
Workout avg speed	V	V	Workout average speed in pre-defined time window
Workout avg power	V	V	Workout average power produced (in Watts) in pre-defined time window
Workout avg norm power	V	V	Workout average normalized power in pre-defined time window
Workout total energy	V	V	Workout total energy in pre-defined time window; considering power produced and workout duration
Workout total elevation gain	V	V	Workout total elevation gain in pre-defined time window; the elevation gain is the total amount cyclist climbs in a workout
Workout total elevation loss	V		Workout total elevation loss in pre-defined time window; the elevation loss is the total amount cyclist descends in a workout
Workout avg elevation	V		Workout average elevation in pre-defined time window
Workout avg temp	V	V	Workout average temperature in pre-defined time window
Workout avg cadence	V	V	Workout average cadence in pre-defined time window; cadence is the rate at which a cyclist pedals
Workout count	V	V	Number of workouts in pre-defined time window
Workout count 28 deg	V	V	Number of workouts in pre-defined time window; temperature above 28 degrees
Workout avg calories last year avg difference	V	V	Difference in workout calories mean in pre-defined time window from annual mean

Table 2. Training Peaks and STRAVA Cyclist features

Feature	TP	STRAVA	Description
Workout avg elevation gain last year avg difference	V	V	Difference in workout elevation gain mean in pre-defined time window from annual mean
Workout avg power last year avg difference	V	V	Difference in workout power mean in pre-defined time window from annual mean
Workout avg elevation loss last year avg difference	V		Difference in workout elevation loss mean in pre-defined time window from annual mean
Workout avg elevation last year avg difference	V		Difference in workout elevation mean in pre-defined time window from annual mean
Workout avg duration last year avg difference	V	V	Difference in workout duration mean in pre-defined time window from annual mean
Workout avg temp last year avg difference	V	V	Difference in workout duration mean in pre-defined time window from annual mean
Workout avg distance last year avg difference	V	V	Difference in workout distance mean in pre-defined time window from annual mean
Workout avg TSS last year avg difference	V		Difference in workout TSS mean in pre-defined time window from annual mean
Workout avg IF last year avg difference	V		Difference in workout IF mean in pre-defined time window from annual mean
Workout avg intensity last year avg difference		V	Difference in workout intensity mean in pre-defined time window from annual mean
Workout avg training load last year avg difference		V	Difference in workout training load mean in pre-defined time window from annual mean
Workout avg speed last year avg difference	V	V	Difference in workout speed mean in pre-defined time window from annual mean
Workout avg cadence last year avg difference	V	V	Difference in workout cadence mean in pre-defined time window from annual mean
Workout avg hr last year avg difference	V	V	Difference in workout heart rate mean in pre-defined time window from annual mean
Workout avg norm power last year avg difference	V	V	Difference in workout normalized power mean in pre-defined time window from annual mean

Table 2. Training Peaks and STRAVA Cyclist features

Feature	TP	STRAVA	Description
Workout avg energy last year avg difference	V	V	Difference in workout energy mean in pre-defined time window from annual mean
Workout last location distance from race		V	Distance in kilometers from the last workout to the upcoming race
Workout avg cadence	V	V	Workout average cadence in pre-defined time window; cadence is the rate at which the cyclist pedals
Workout count	V	V	Number of workouts in pre-defined time window
Workout count >28 deg	V	V	Number of workouts in pre-defined time window; temperature was above 28 degrees
Workout avg calories last year avg difference	V	V	Difference in workout calories mean in pre-defined time window from annual mean
Workout avg elevation gain last year avg difference	V	V	Difference in workout elevation gain mean in pre-defined time window from annual mean
Workout avg power last year avg difference	V	V	Difference in workout power mean in pre-defined time window from annual mean
Workout avg elevation loss last year avg difference	V		Difference in workout elevation loss mean in pre-defined time window from annual mean
Workout avg elevation last year avg difference	V		Difference in workout elevation mean in pre-defined time window from annual mean
Workout avg duration last year avg difference	V	V	Difference in workout duration mean in pre-defined time window from annual mean
Workout avg temp last year avg difference	V	V	Difference in workout duration mean in pre-defined time window from annual mean
Workout avg distance last year avg difference	V	V	Difference in workout distance mean in pre-defined time window from annual mean
Workout avg TSS last year avg difference	V		Difference in workout TSS mean in pre-defined time window from annual mean
Workout avg IF last year avg difference	V		Difference in workout IF mean in pre-defined time window from annual mean
Workout avg intensity last year avg difference		V	Difference in workout intensity mean in pre-defined time window from annual mean

Table 2. Training Peaks and STRAVA Cyclist features

Feature	TP	STRAVA	Description
Workout avg training load last year avg difference		V	Difference in workout training load mean in pre-defined time window from annual mean
Workout avg speed last year avg difference	V	V	Difference in workout speed mean in pre-defined time window from annual mean
Workout avg cadence last year avg difference	V	V	Difference in workout cadence mean in pre-defined time window from annual mean
Workout avg hr last year avg difference	V	V	Difference in workout heart rate mean in pre-defined time window from annual mean
Workout avg norm power last year avg difference	V	V	Difference in workout normalized power mean in pre-defined time window from annual mean
Workout avg energy last year avg difference	V	V	Difference in workout energy mean in pre-defined time window from annual mean
Workout last location distance from race		V	Distance in kilometers from the last workout to the current race

Table 3. Pro-Cycling Stats Race features

Feature	Race	Stage	Description
Race difficulty level	V	V	Race difficulty level (e.g., Hard, Intermediate, Easy)
Race continent	V	V	Race continent
Stage ranking	AVG	V	Ranking of stage difficulty level
Stage profile score	AVG	V	Score of the stage that indicates the race profile hardness
Stage profile type	AVG	V	Stage profile type (i.e., hilly, mountainous, flat)
Stage distance	AVG	V	Distance of stages in a race
Stage elevation gain	AVG	V	Elevation gain of stages in a race
Stage temp avg	AVG	V	Temperature of stages in a race
Race classification	V	V	Race class (i.e., WorldTour 1.WT)
Race total stage distance	V	V	Race stage distance sum
Race total stage elevation gain	V	V	Race stage elevation gain sum
Race number of stage	V	V	Number of stage in the race
Race has flat profile	V	V	Race is mostly flat

Table 3. Pro-Cycling Stats Race features

**Feature**	**Race**	**Stage**	**Description**
Race has hills and flat finish	V	V	Race profile is hilly but flat in the end
Race has hilly profile	V	V	Race profile is hilly
Race has mountains and hilly finish	V	V	Mountainous race with hilly end
Race has mountain flat finish	V	V	Mountainous race, flat end
