# Supplementary material for: Pro-cycling team cyclist assignment for an upcoming race
Source: PLoS One. 2024 Mar 4;19(3):e0297270. doi: 10.1371/journal.pone.0297270 (PMC10911621; doi:10.1371/journal.pone.0297270)
Supplement: S2 Appendix — Full results of Experiment 3,4 for Israel-Premier Tech, Groupama-FDJ, and Team Jumbo-Visma. (PDF) [file pone.0297270.s002.pdf]

Appendix 2 - Full Results - Experiments 3,4

Here you can see a comprehensive presentation of experiment 3 and 4 results, focusing on the detailed performance outcomes for each of the the teams: Israel-Premier Tech, Groupama-FDJ, and Team Jumbo-Visma. In contrast to the condensed averages presented in the main paper, this appendix offers an understanding of individual team performances.

Experiment 3—Cyclist–Race all teams on STRAVA

Since in the previous experiments it was seen that the performance was quite similar whether using TP or STRAVA, we extend our experiments to two more pro-cycling teams, Jumbo-Visma and Groupma-FDJ, whose TP data was not accessible. We describe here the use of the cyclist–race modeling results after applying them to each of the teams, and their average to demonstrate the general performance. Fig. 1 presents the performance of recent workouts over multiple time windows, represented in each curve using the cyclist–race model, in comparison to the baselines. The charts for all the teams perform quite similarly, while for IPT, Fig. 1a, the performance at 7 weeks is quite better than the others. For Groupama-FDJ, Fig. 1b, the 3-week window performs better than the others, and for Jumbo-Visma, Fig. 1c, on average, 1d the performance is quite the same for any of the time windows. In all charts, the use of RaceFit outperforms the popularity baselines. Note that these results are the mean of multiple modeling choices, including the use of not using imputation and data removal, as well as several classifiers, whose results are relatively not so good, but this does show the overall effect of using several weeks. In the later experiments, we will show the results with the best performing values.

Fig. 2 shows the performance when using imputation or not, which clearly shows that using imputation was much better for all the teams. However, without imputation, the performance is still better than that of the popularity baselines.

Fig. 3 shows the performance using several classifiers, in which all perform better than the baselines, while the use of the CatBoost classifier performs meaningfully better in both metrics. Random Forest is second best after CatBoost for all teams, and for IPT, the third-best classifier is Logistic Regression, followed by Decision Tree. For Groupama-FDJ and Jumbo-Visma teams and on average for all teams, the Decision Tree classifier is slightly better than Logistic Regression.

Fig. 4 shows the $recall@(n+k)$, in which n is the number of cyclists who are required to participate in an upcoming race and k is the number of spare cyclists the system recommends for the upcoming race. We see that when requesting the exact number of required cyclists (k = 0) for the upcoming race, CatBoost correctly recommends about 50% of the cyclists who in fact participated in the race. In all charts, the CatBoost and Random Forest classifiers perform similarly. However, in Fig. 4a, for all k, Logistic Regression performs better than the Decision Tree. In Fig. 4b, the Decision Tree performs better than Logistic Regression for most of the k values, and in Figs. 4c and 4d, the same performance is shown, but for values of k smaller than four.

Fig. 5 shows the performance of the best-performing parameters on average for all teams, based on the previous analyses. The best time window size on average over all the teams is 3 weeks, the threshold which performs best is the 40% missing value dropping ratio, use of imputation improves the results, and CatBoost outperforms the other classifiers. The parameter results on average over the teams are not identical to the best-performing parameters for each team. Fig. 5a, for example, shows the results of IPT, in which the best-performing parameters are a time window of 7 weeks and a threshold of 60%. In Fig. 5c of Jumbo-Visma, the best-performing time window is 7 weeks. For Groupama-FDJ, the best-performing parameters are identical to those which

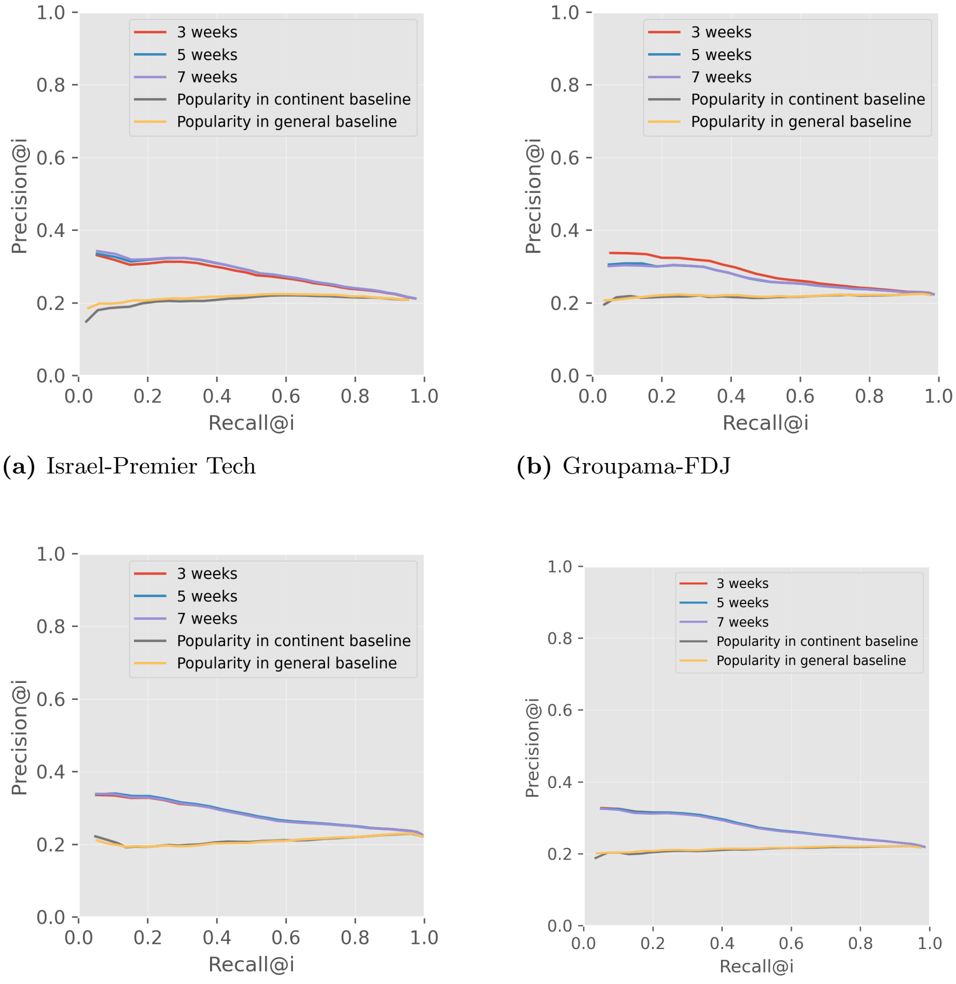

(a) Israel-Premier Tech

(b) Groupama-FDJ

(c) Team Jumbo-Visma

(d) Average of all teams

Fig 1. Comparison of different numbers of weeks of workout time windows for each team, and their average performance. The different time windows perform quite similarly, and better than the baselines.

were chosen on average. However, while the differences in the parameter values are not that meaningful in most previous analyses, in the next figure, we present the performance of each team according to their best parameters' values.

Fig. 6 presents the model's parameters with the best-performing values for each team individually. For IPT, the best time window size is 7 weeks, while for Jumbo-Visma, it is the 5-week window. The best time window of Groupama-FDJ is 3 weeks as, on average, for all the teams. The threshold for Figs. 6b and 6c is 40%, like the threshold chosen to best perform in the average result graphs. However, in the IPT experiments, the best-performing threshold is 60%.

Experiment 4—Cyclist–Stage all teams on STRAVA

We describe here the results of the cyclist–stage experiment on the various teams' on STRAVA data. Fig. 7 presents the performance of the different time windows in weeks.

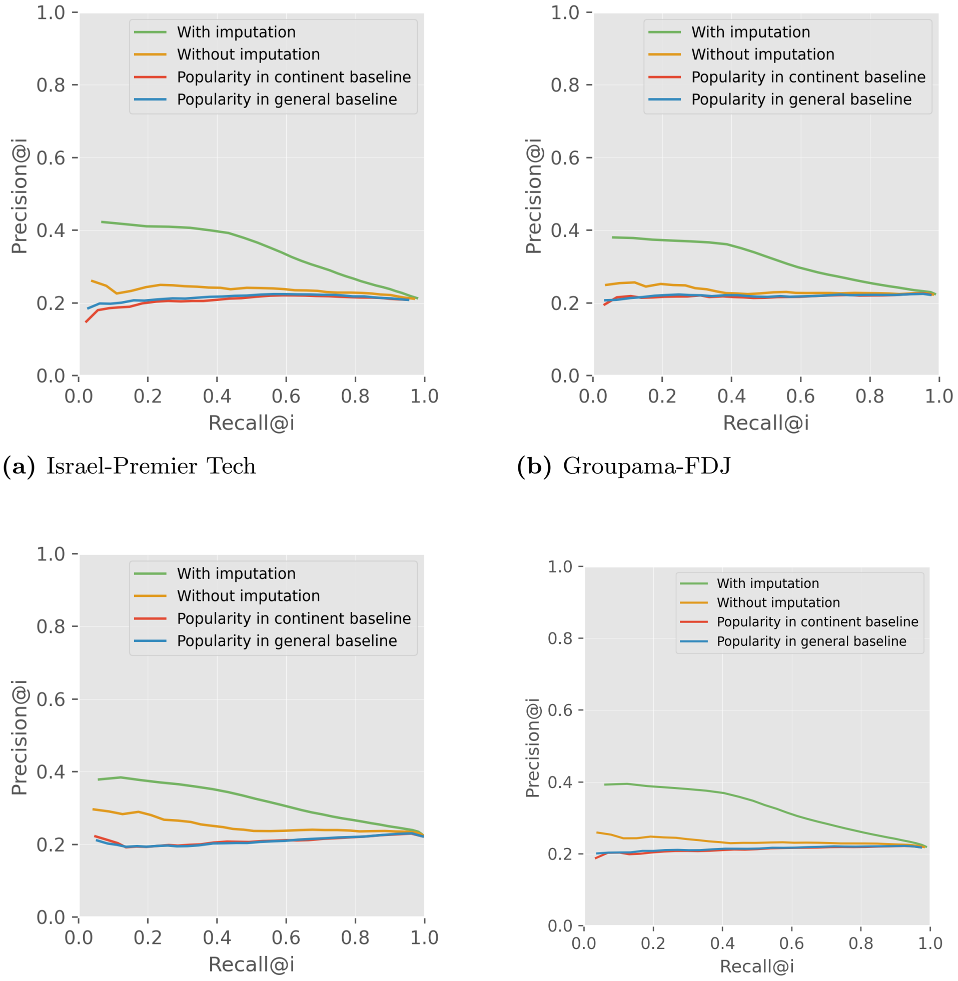

(a) Israel-Premier Tech **(b)** Groupama-FDJ

(c) Team Jumbo-Visma **(d)** Average of all teams

Fig 2. The use of imputation performs clearly better than without imputation.

For all the teams, the use of a 5-week time window yields the best performance. However, for IPT, the next best time window is 3 weeks, while for the other teams, on average, the 3-week window performs worse than the 7-week window. For all time windows, RaceFit performs better than the popularity baselines.

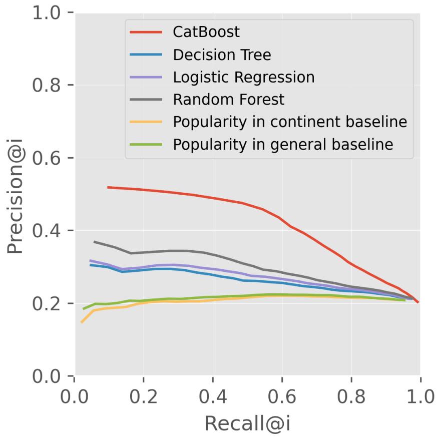

(a) Israel-Premier Tech

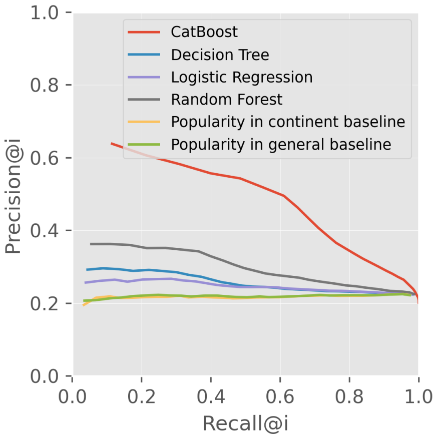

(b) Groupama-FDJ

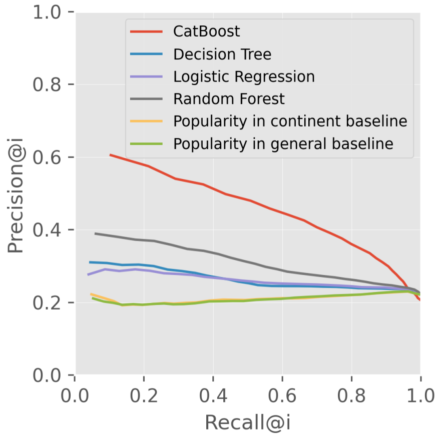

(c) Team Jumbo-Visma

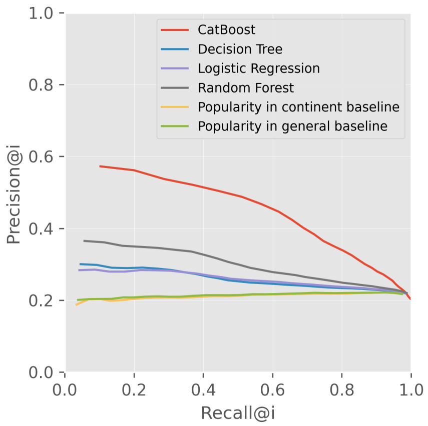

(d) Average of all teams

Fig 3. CatBoost performs best in all cases, and RaceFit performs better than the baselines.

Fig. 8 shows the advantage of using imputation. In all of the teams, the use of imputation improves performance. However, even without imputation, the performance is better than the popularity baselines.

In Fig. 9, the results of the classifiers that were evaluated are shown. For all the teams, the CatBoost classifier outperforms the others. However, the performance for IPT (Fig. 9a) seems much better than for the other teams. On average and in Figs. 9b and 9c, Random Forest is the next best performing classifier, while for IPT (Fig. 9a), Random Forest performs similarly to Logistic Regression.

Fig. 10 shows the $recall@(n+k)$ for the cyclist–stage version of RaceFit, in which n is the actual number of cyclists recommended and k is the number of spare cyclists recommended, for the coach to choose from if needed. The CatBoost classifier yields the best performance for all of the teams, followed by Random Forest. For IPT, the classifier following Random Forest is the Logistic Regression classifier and then Decision Tree for each k, but for Groupama-FDJ, Decision Tree performs slightly better. In Fig. 10c of Jumbo-Visma, for most of the k values Decision Tree performs somewhat better,

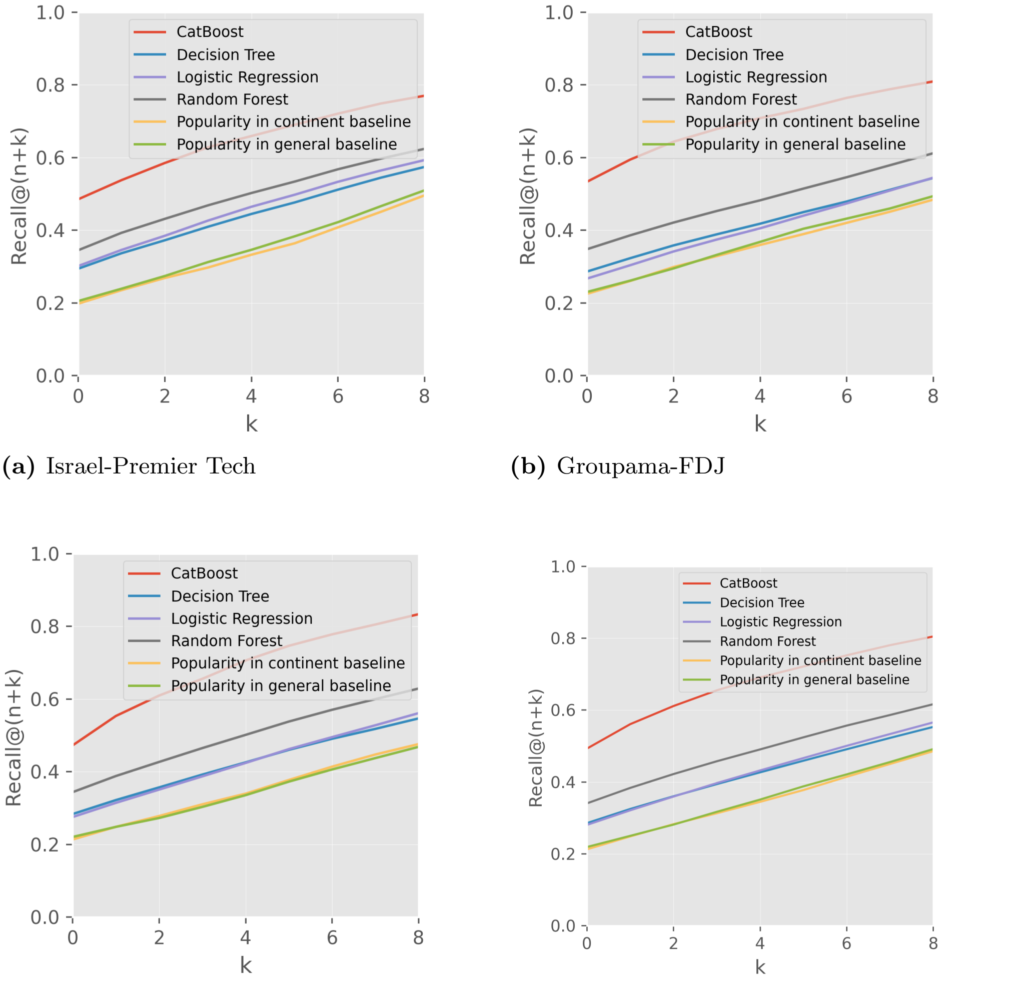

(a) Israel-Premier Tech **(b)** Groupama-FDJ

(c) Team Jumbo-Visma **(d)** Average of all teams

Fig 4. Classifier parameter comparison of the different teams. CatBoost performs best, in all cases, RaceFit performs better than the baselines.

and for k greater than 4, Logistic Regression performs better. For all the teams, the baselines perform more poorly than the RaceFit algorithm.

Fig. 11 presents the best-performing parameters on average over all teams' results. The best performance achieved by window size is the 5-week window, the best threshold on average is the 60% missing value dropping ratio, use of imputation to fill missing values, and CatBoost as a classifier. The only team with different best performance parameters is Groupama-FDJ, which has a threshold of 40% as the best-performing threshold.

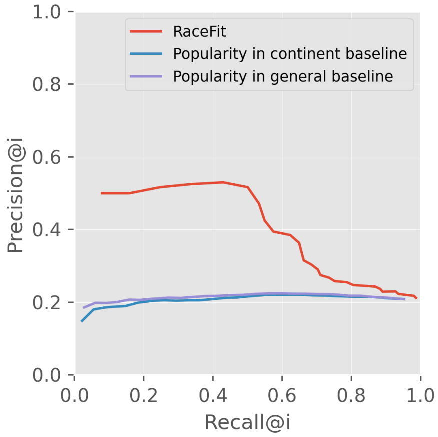

(a) Israel-Premier Tech

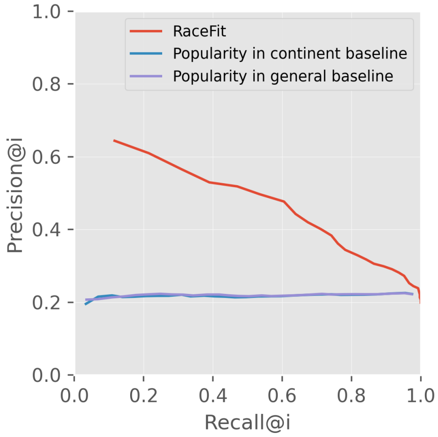

(b) Groupama-FDJ

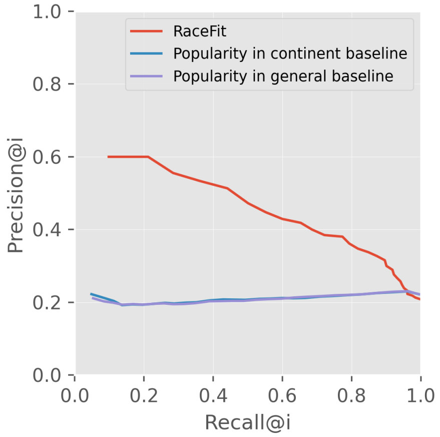

(c) Team Jumbo-Visma

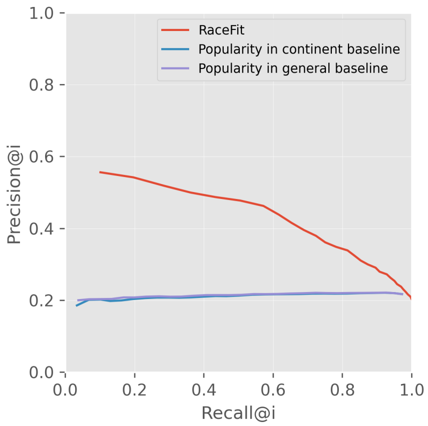

(d) Average of all teams

Fig 5. RaceFit with the best parameters for each of the teams, and their average, shows that it is much better than the popularity baselines.

Fig. 12 shows the results of the best parameters chosen for each oteam individually. The best time window for all the teams is the 5-week window. The best threshold is 60% for all the teams, except for Groupama-FDJ (Fig. 12b), which is 40%. The best classifier for all teams is CatBoost, and use of imputation was effective for all as well. Looking at the results in Fig. 11 and the parameters that worked best for all teams on average, the results perform quite similarly; thus, it is reasonable to use the parameter values that work for all teams on average.

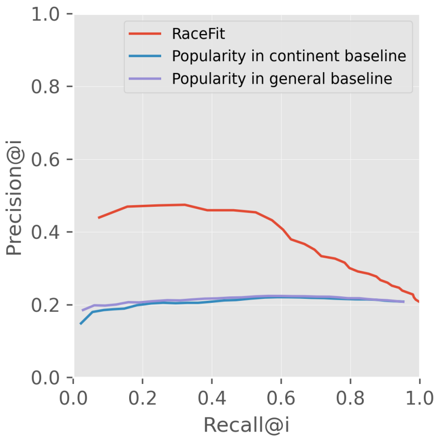

(a) Israel-Premier Tech

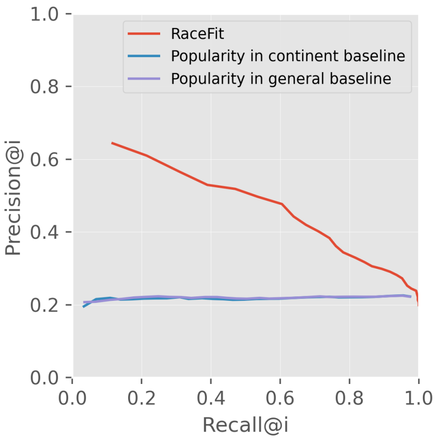

(b) Groupama-FDJ

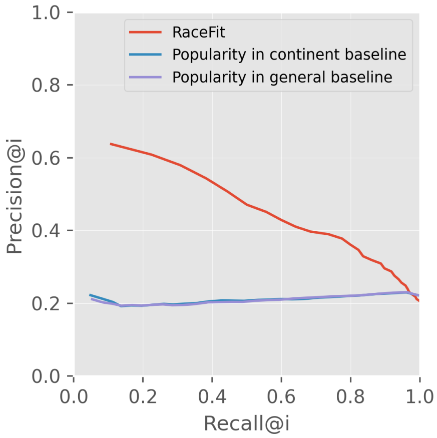

(c) Team Jumbo-Visma

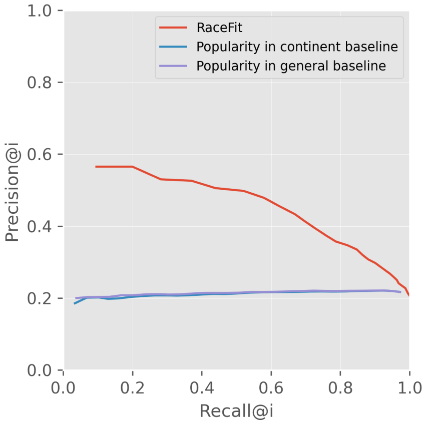

(d) Average of all teams

Fig 6. Comparison of the best parameters for each team individually.

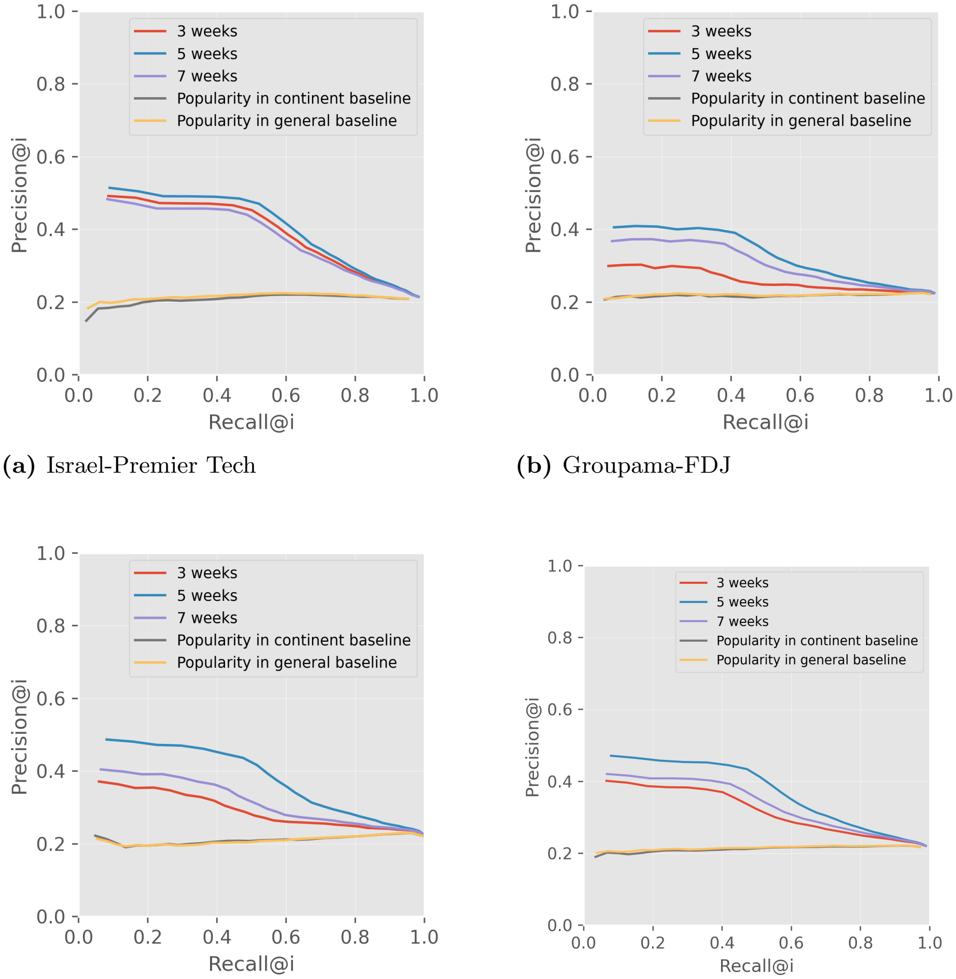

(a) Israel-Premier Tech

(b) Groupama-FDJ

(c) Team Jumbo-Visma

(d) Average of all teams

Fig 7. Using 5 weeks performs best, and the different time windows perform better than the baselines.

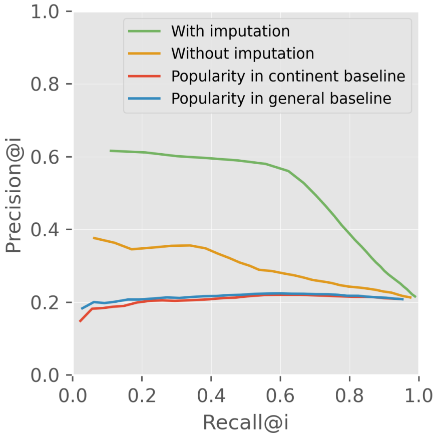

(a) Israel-Premier Tech

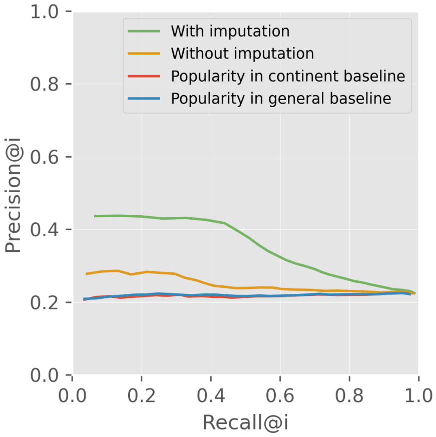

(b) Groupama-FDJ

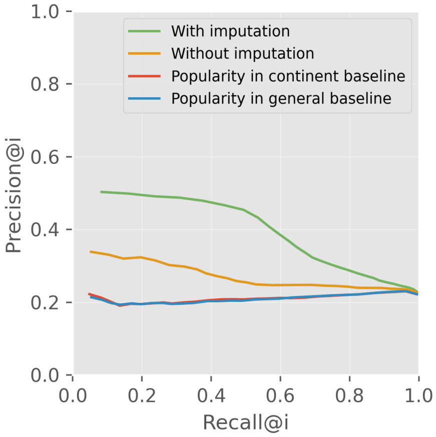

(c) Team Jumbo-Visma

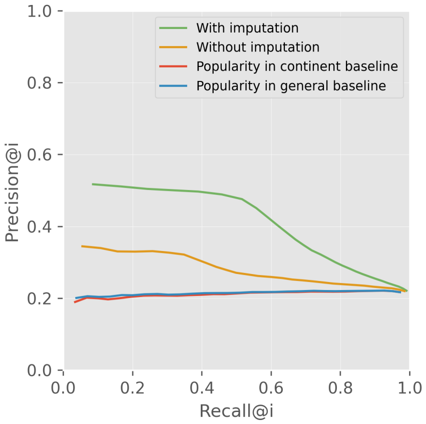

(d) Average of all teams

Fig 8. RaceFit with and without imputation. The use of imputation improves the results for all the teams.

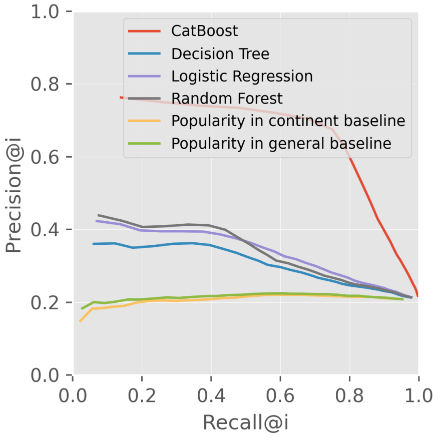

(a) Israel-Premier Tech

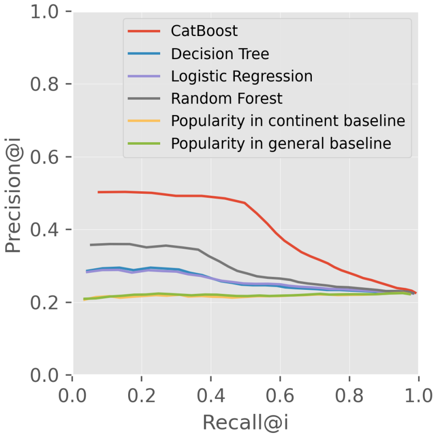

(b) Groupama-FDJ

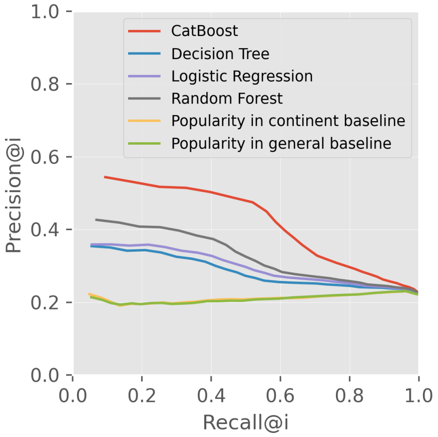

(c) Team Jumbo-Visma

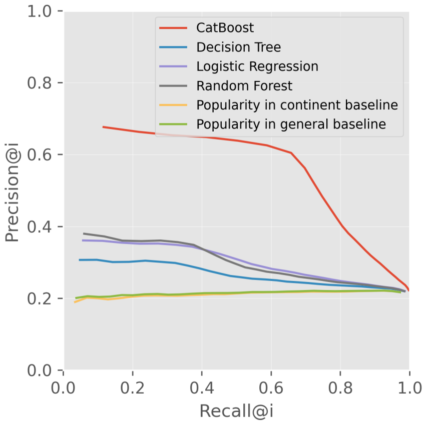

(d) Average of all teams

Fig 9. The CatBoost classifier outperforms the other classifiers, and with IPT, the performance is much higher.

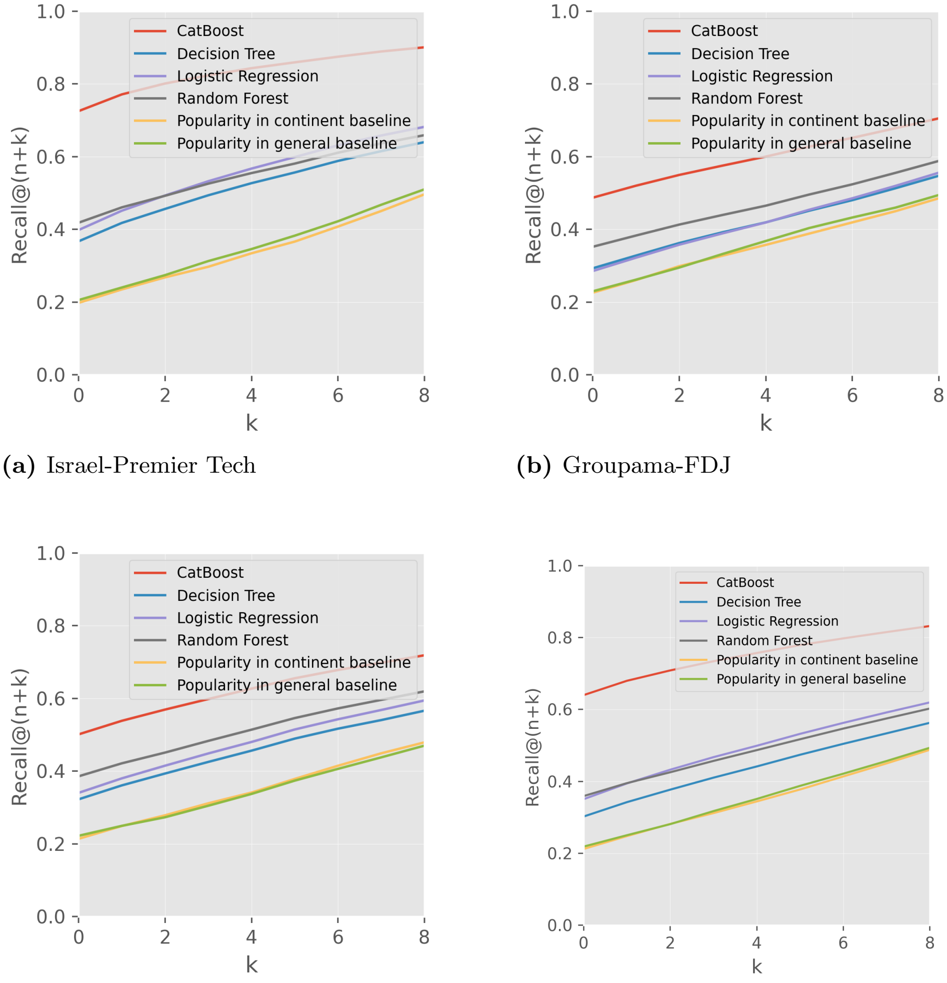

(a) Israel-Premier Tech

(b) Groupama-FDJ

(c) Team Jumbo-Visma

(d) Average of all teams

Fig 10. The CatBoost classifier outperforms the others, while all the classifiers perform better than the baselines.

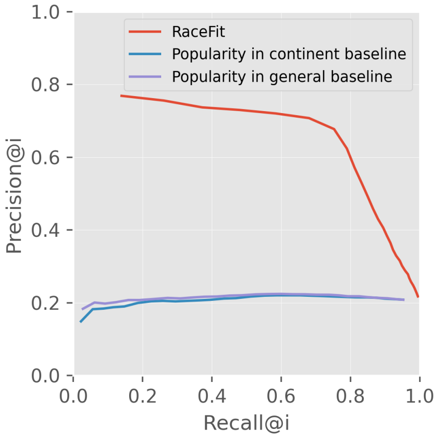

(a) Israel-Premier Tech

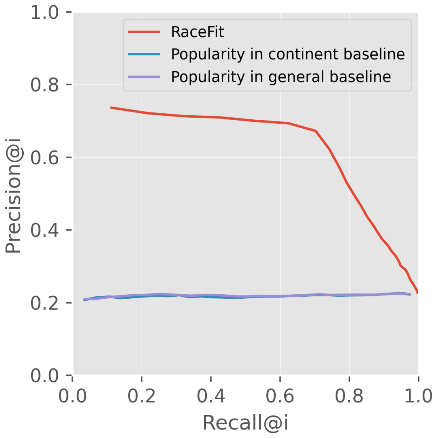

(b) Groupama-FDJ

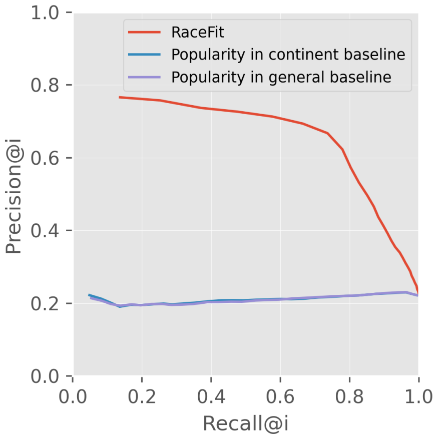

(c) Team Jumbo-Visma

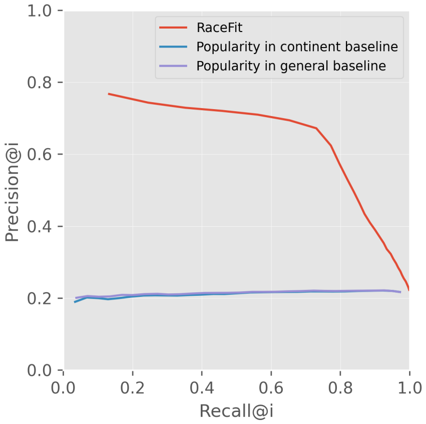

(d) Average of all teams

Fig 11. Comparison of the best parameters on average of all teams.

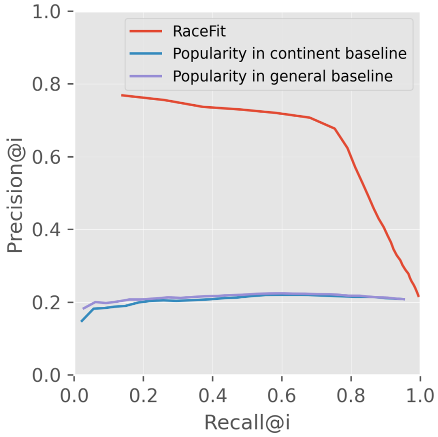

(a) Israel-Premier Tech

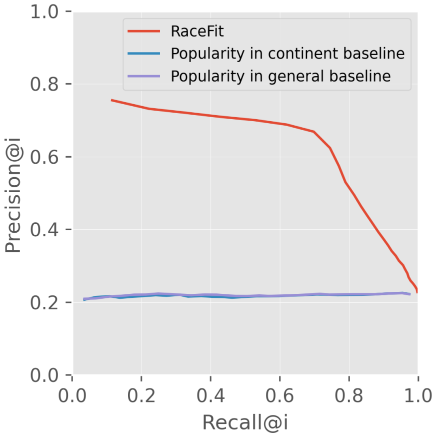

(b) Groupama-FDJ

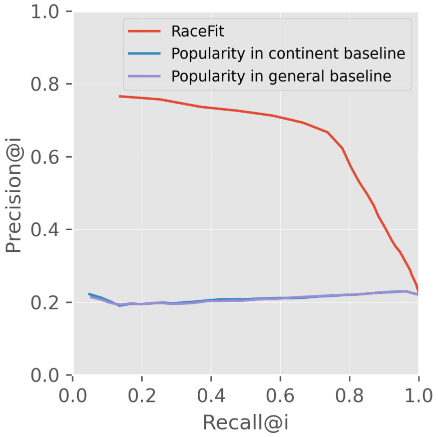

(c) Team Jumbo-Visma

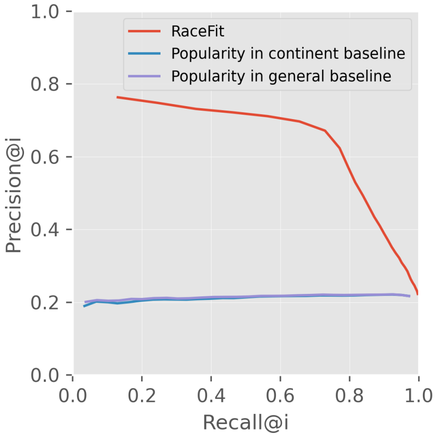

(d) Average of all teams

Fig 12. The performance for each team with its best parameter values show quite similar performance for all the teams.
