# Supplementary material for: Pro-cycling team cyclist assignment for an upcoming race
Source: PLoS One. 2024 Mar 4;19(3):e0297270. doi: 10.1371/journal.pone.0297270 (PMC10911621; doi:10.1371/journal.pone.0297270)
Supplement: S3 Appendix — Results of the feature importance analysis with description. (PDF) [file pone.0297270.s003.pdf]

Appendix 3 - Feature importance

We employed two approaches to identify the most important features used: data-driven methods - Relief, Information Gain, and Chi-Square, and the CatBoost's or SHAP feature importance. We discuss the feature importance results while distinguishing three groups: cyclist features, workout features, and race features. In the Information Gain (IG) method (Fig. 1), for all teams, cyclist features have the highest importance. Specifically, the number of weeks that passed since the last race the cyclist raced till the upcoming race, and the physical distance from the last race the cyclist raced to the upcoming race location. The next most important features are the workout features, and more specifically, the difference between the cyclist's average performance in the last year to performance in the last 5 weeks. Note that there are no race features in the top 10 most important features in the IG method. In the Relief method results (Fig. 2), for all teams, the three features groups have representation in the top-10 most influential features. The distance from the last race a cyclist raced that we saw in the top features of the IG method, appears in the top 10. In these figures, workout features are influential along with the race features. The race features like the total distance and elevation gain are in the top-10 most important features which makes sense while different race profiles fit different cyclists' skills like sprinting or climbing. The Chi-Square method (Fig. 3) is similar to the Relief method in the features group distribution. However, the importance values change drastically compared to the Relief method's incremental changes. The cyclist distance from the last race and the race total distance have high impact such as in the last figure. CatBoost feature importance in Fig. 4 shows a strong impact of the distance and time constraints as in the IG results. Although, in the CatBoost results, the race features are the next most important, and workout features do not appear in the top 10 at all. The SHAP method results are quite similar to those ofCatBoost, which makes sense as both methods are incorporated with the modeling process. An additional cyclist feature, in addition to the constraint features in all teams, is the cyclist race rate, which refers to the cyclist's race frequency in the particular team.

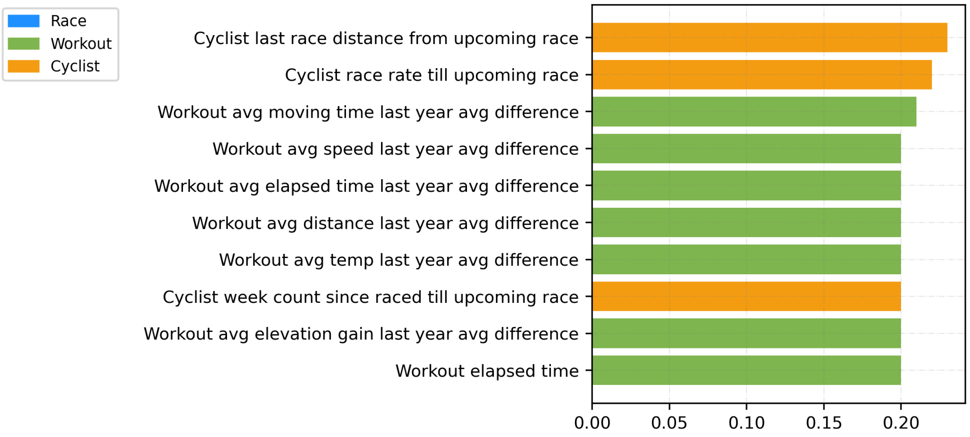

(a) Israel-Premier Tech

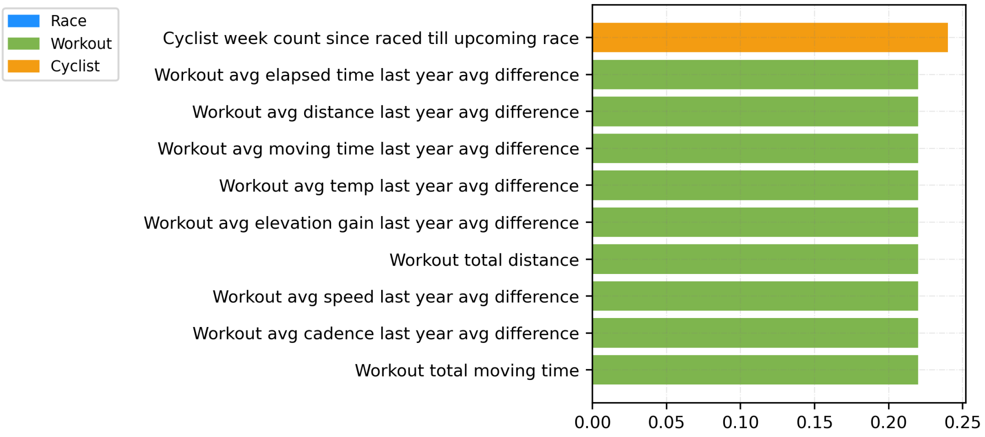

(b) Groupama-FDJ

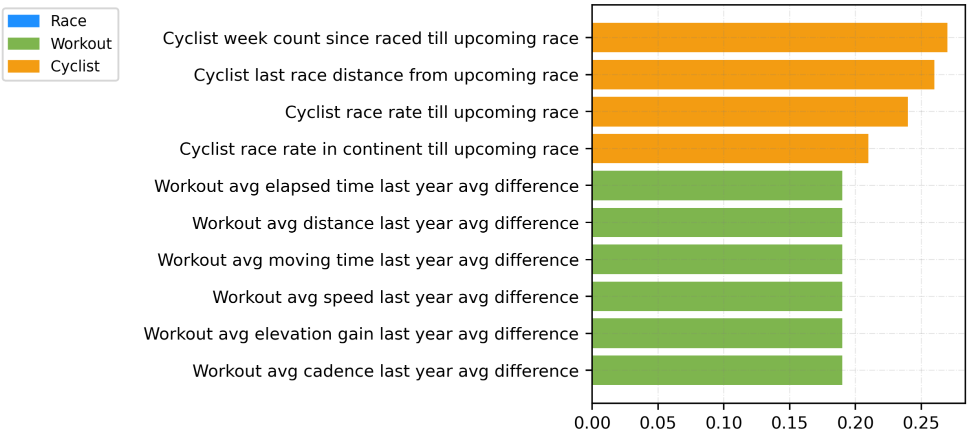

(c) Team Jumbo-Visma

Fig 1. Feature importance based on Information Gain method

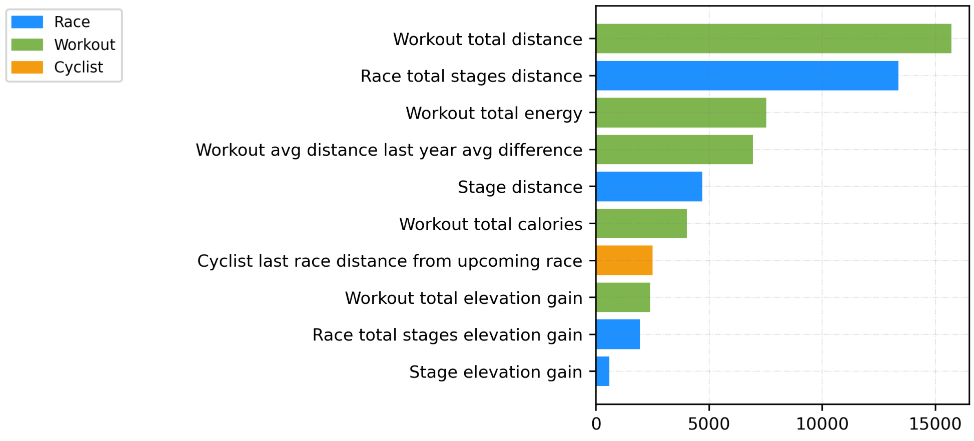

(a) Israel-Premier Tech

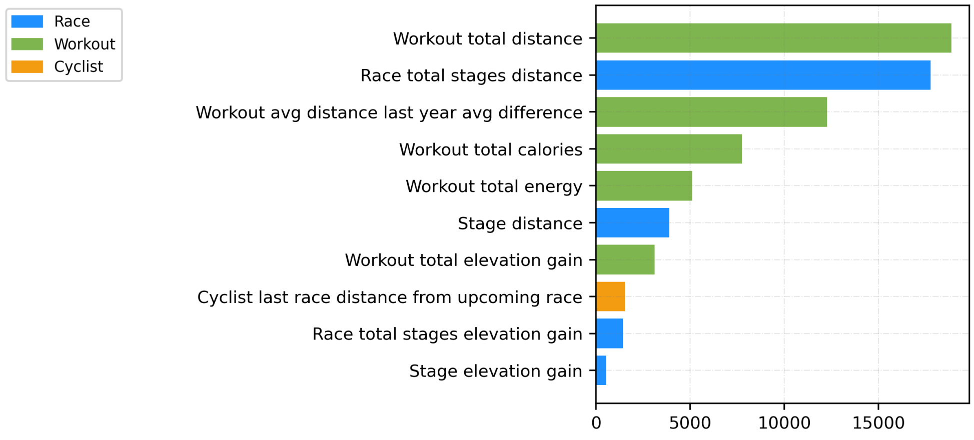

(b) Groupama-FDJ

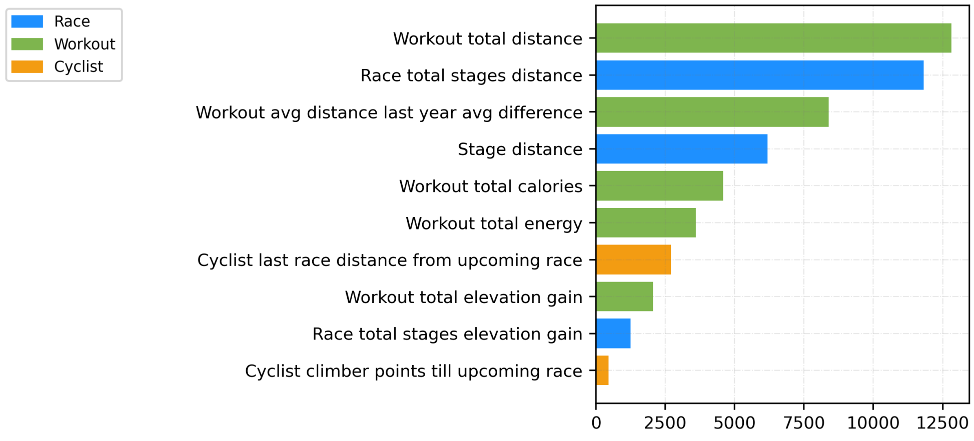

(c) Team Jumbo-Visma

Fig 2. Feature importance based on the Relief method.

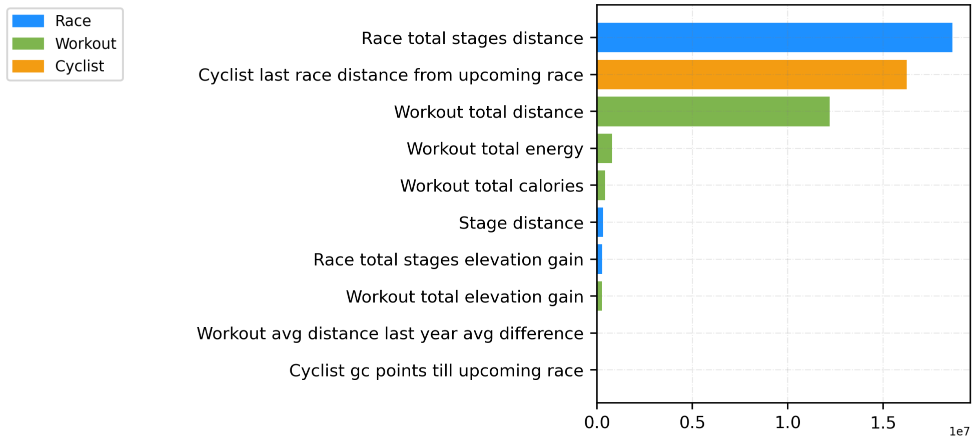

(a) Israel-Premier Tech

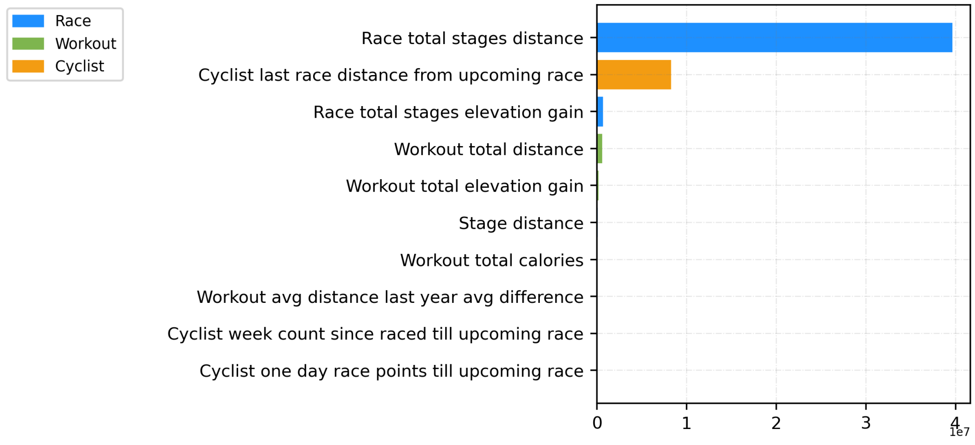

(b) Groupama-FDJ

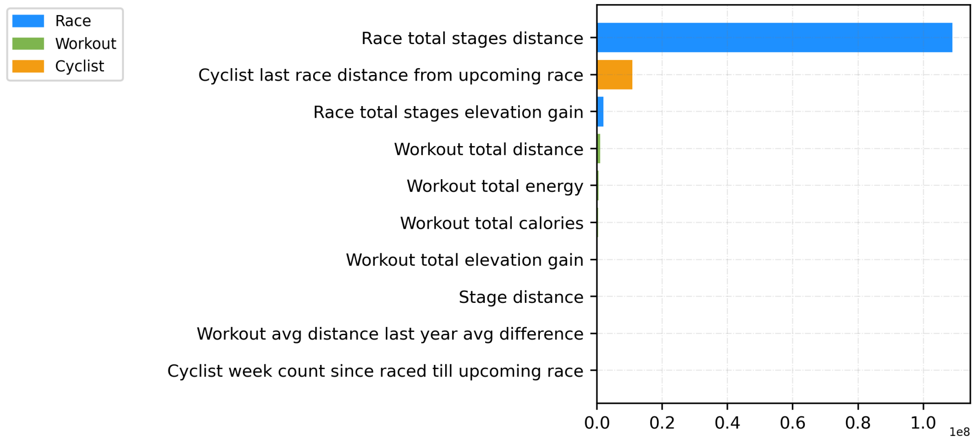

(c) Team Jumbo-Visma

Fig 3. Feature importance based on the Chi-Square method.

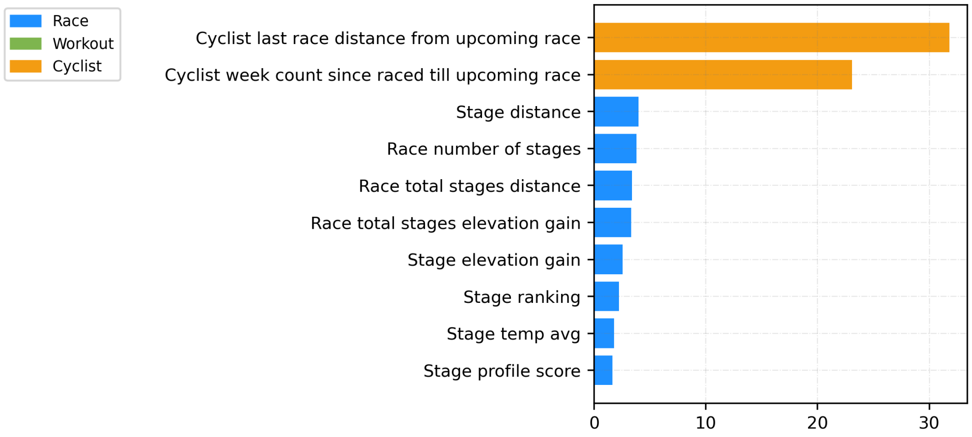

(a) Israel-Premier Tech

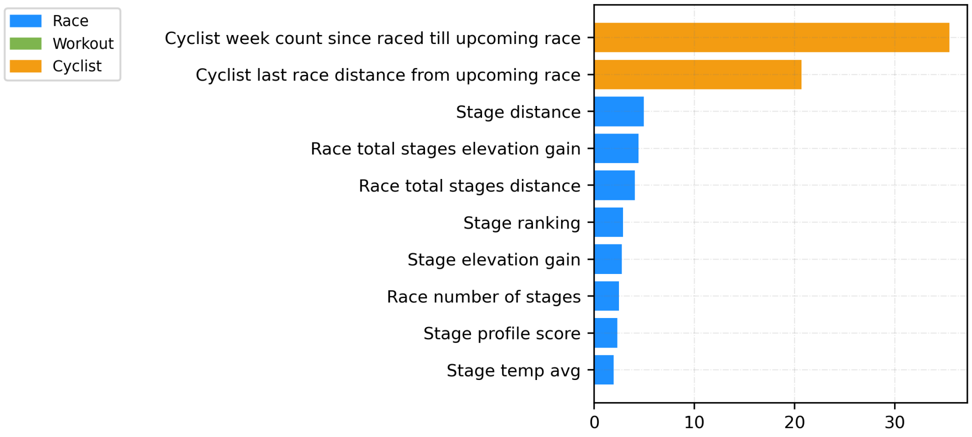

(b) Groupama-FDJ

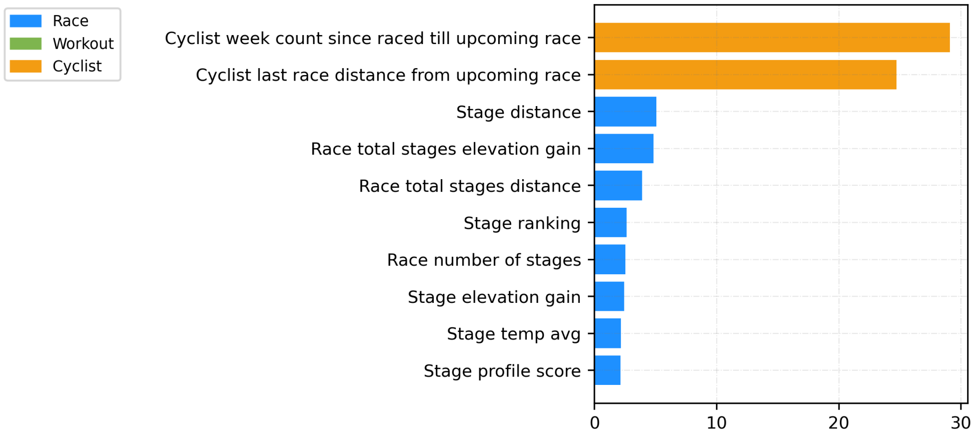

(c) Team Jumbo-Visma

Fig 4. Feature importance based on the CatBoost method.

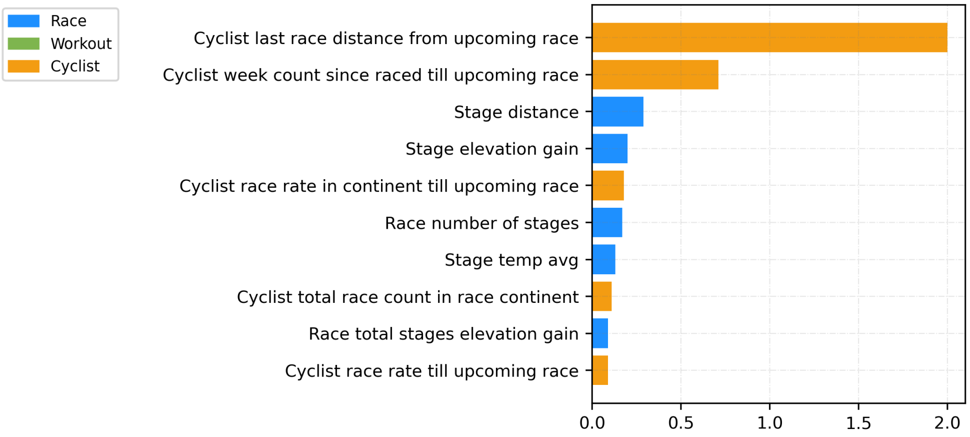

(a) Israel-Premier Tech

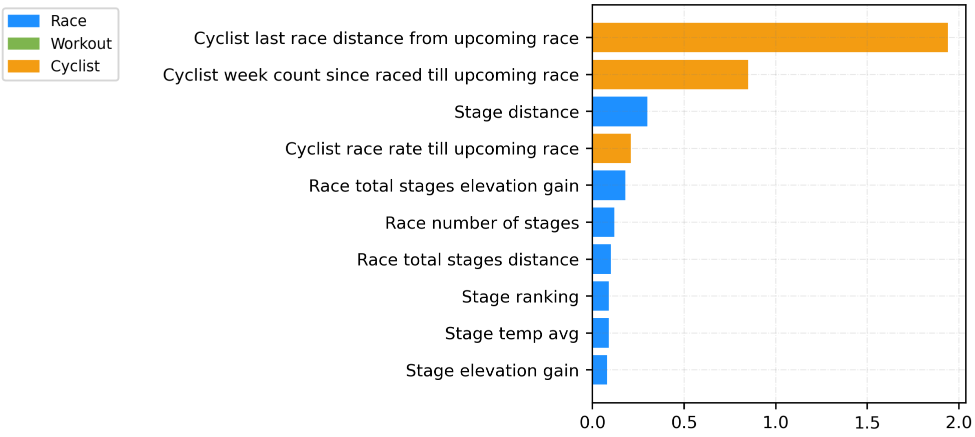

(b) Groupama-FDJ

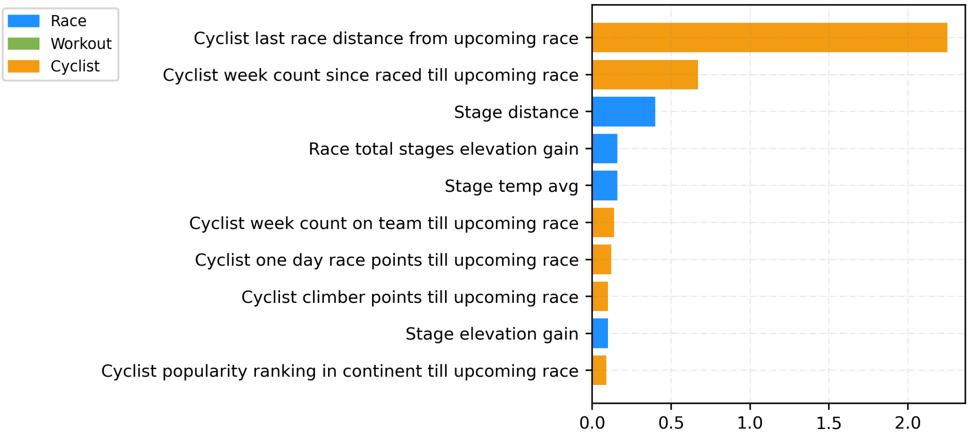

(c) Team Jumbo-Visma

Fig 5. Feature importance based on the SHAP method.
